# Systematic Development and Characterization of Novel, High Drug-Loaded, Photostable, Curcumin Solid Lipid Nanoparticle Hydrogel for Wound Healing

**DOI:** 10.3390/antiox10050725

**Published:** 2021-05-05

**Authors:** Simarjot Kaur Sandhu, Suneel Kumar, Jayant Raut, Mandeep Singh, Sandeep Kaur, Garima Sharma, Tomas L. Roldan, Sonia Trehan, Jennifer Holloway, Gabriella Wahler, Jeffrey D. Laskin, Patrick J. Sinko, Francois Berthiaume, Bozena Michniak-Kohn, Praveen Rishi, Narayanan Ganesh, Indu Pal Kaur

**Affiliations:** 1 Institute of Pharmaceutical Sciences, Panjab University, Chandigarh 160014, India; simarjots@gmail.com (S.K.S.); rautjayant123@gmail.com (J.R.); mandysidhu19@hotmail.com (M.S.); kaursandeep9987@gmail.com (S.K.); garimasharma194@gmail.com (G.S.); 2 Department of Biomedical Engineering, Rutgers University, Piscataway, NJ 08854, USA; sk1350@soe.rutgers.edu (S.K.); fberthia@soe.rutgers.edu (F.B.); 3 Department of Pharmaceutics, Ernest Mario School of Pharmacy, Rutgers University, Piscataway, NJ 08854, USA; troldan242@gmail.com (T.L.R.); borgia.holloway@gmail.com (J.H.); sinko@rutgers.edu (P.J.S.); michniak@pharmacy.rutgers.edu (B.M.-K.); 4 Counter ACT Center of Excellence, Rutgers University, Piscataway, NJ 08854, USA; gabrieco@pharmacy.rutgers.edu (G.W.); jl1450@eohsi.rutgers.edu (J.D.L.); 5 Center for Dermal Research (CDR), Life Sciences Building, Rutgers University, Piscataway, NJ 08854, USA; strehan13@gmail.com; 6 Department of Pharmacology and Toxicology, Rutgers University, Piscataway, NJ 08854, USA; 7 Department of Microbiology, Panjab University, Chandigarh 160014, India; rishipraveen@yahoo.com; 8 Jawaharlal Nehru Cancer Hospital & Research Centre, Bhopal 462001, India; nganeshresearch@gmail.com

**Keywords:** nanocarriers, safety, biofilm, wound closure, oxidative stress, TNFα, VEGF

## Abstract

The study aims to develop high drug-loaded (about 15% lipid matrix) curcumin solid lipid nanoparticles (CSLNs) for wound healing. CSLNs prepared by hot, high-pressure homogenization, without using organic solvents, were optimized using the Taguchi design followed by the central composite design. The optimized CSLNs exhibited a high assay/drug content (0.6% *w*/*w*), solubility (6 × 10^5^ times), and EE (75%) with a particle size < 200 nm (PDI—0.143). The CSLNs were safe (in vitro and in vivo), photostable, autoclavable, stable up to one year at 30 °C and under refrigeration and exhibited a controlled release (zero-order; 5 days). XRD, FTIR, and DSC confirmed solubilization and entrapment of the curcumin within the SLNs. TEM and FESEM revealed a smooth and spherical shape. The CSLNs showed a significant antimicrobial effect (MIC of 64 µg/mL for planktonic cells; 512 µg/mL for biofilm formation; and 2 mg/mL for mature biofilm) against *Staphylococcus aureus* 9144, while free curcumin dispersion did not exhibit any effect. This is the first report on the disruption of mature biofilms by curcumin solid lipid nanoparticles (CSLNs). The cell proliferation potential of CSLNs was also evaluated in vitro while the wound healing potential of CSLNs (incorporated in a hydrogel) was assessed in vivo. In (i) nitrogen mustard gas and (ii) a full-thickness excision wound model, CSLNs exhibited (a) significantly faster wound closure, (b) histologically and immunohistochemically better healing, (c) lower oxidative stress (LPO) and (d) inflammation (TNFα), and (e) increased angiogenesis (VEGF) and antioxidant enzymes, i.e., catalase and GSH levels. CSLNs thus offer a promising modern wound therapy especially for infected wounds, considering their effects in mature biofilm disruption.

## 1. Introduction

Wound healing is a complex biological process composed of interrelated and overlapping phases of homeostasis, inflammation, migration, proliferation, and maturation [1]. The wound bed provides an ideal environment for microbial growth, facilitating the penetration of pathogens into the underlying tissue, with a potential for hematogenous dissemination. Further, inflammation followed by oxidative stress, mediated by the formation of free radicals, leads to DNA breakage, lipid peroxidation and enzyme inactivation resulting in impaired wound healing. Currently employed antimicrobial agents possess limited utility due to toxicity, narrow antimicrobial coverage, inadequate wound bed penetration, and growing bacterial resistance [2,3,4]. In addition, mainline treatments such as silver sulfadiazine may delay burn wound healing as it is toxic to regenerating keratinocytes [5].

An optimum wound healing dressing or agent should thus (i) reduce inflammation, (ii) protect the wound tissue from bacterial infection, and (iii) induce cell proliferation to aid in the reconstruction of damaged tissue [6]. It should ideally also act as (iv) an antioxidant, as free radicals are considered as the major cause of inflammation during the wound healing process [7]. Curcumin, chemically known as diferuloylmethane or 1,7-bis(4-hydroxy-3-methoxyphenyl)-1,6-heptadiene-3,5-dione, is a low-molecular-weight polyphenolic phytoconstituent obtained from the powdered rhizome of Curcuma longa L. It has been used as an oral supplement in various medical and cutaneous conditions [8]. The wound healing potential of curcumin is attributed to its multi-pharmacological effects such as its anti-inflammatory [9], anti-infectious [10,11], analgesic, [12] and antioxidant [13,14] nature. Curcumin has also been reported to augment cutaneous wound healing through tissue remodeling, granulation tissue formation, and collagen deposition [7,15]. The pleiotropic activities of curcumin target various phases of the healing process, resulting in accelerated wound closure and restoration of healed tissue functionality. Furthermore, curcumin has been shown to provide significant protection against hydrogen peroxide-induced damage to human keratinocytes and human dermal fibroblasts [16]. A decrease in the levels of lipid peroxides (LPs), while an increase in the levels of catalase and glutathione peroxidase (GPx), activities on the treatment with curcumin, resulting in accelerated wound healing is also reported [17,18]. The potent antioxidant activity of curcumin is the result of its ability to shift electrons or easily give H-atoms from two methoxy phenol groups. Further, it contains a variety of functional groups, such as β-diketone, and several π electrons that have a prominent electron transfer capability. The phenolic hydroxyl groups of curcumin impart it with the ROS scavenging ability, and the di-ketone structure is considered to be responsible for its ability to bind to metals [19,20].

However, the potential of curcumin for therapeutic translation is hindered due to its poor water solubility, low permeability and low stability (under physiological conditions and photostability) [21]. Another disadvantage of curcumin is that at high concentrations it may manifest pro-oxidant effects resulting in concentration-dependent, cytotoxic, genotoxic, apoptotic, and ROS-generating effects observed even in normal fibroblastic cells (L-929); though the effects were more prominently reported in cancer cells [22]. In an in vitro wound contraction assessment, curcumin was found to significantly increase ROS formation at higher concentrations (25 μM) resulting in fibroblast apoptosis. In contrast, 10–15 μM concentrations resulted in maximal haem oxygenase activity [23], which has an important role in wound healing including diabetic chronic wounds. Hence, a water-soluble curcumin formulation with a controlled release would be most optimal for the clinical healing of wounds [24].

Currently, it was envisaged that the encapsulation of curcumin within a physiological lipid matrix of solid lipid nanoparticles (CSLNs) can simultaneously address the solubility, stability, metabolic degradation, and permeability issues of curcumin in addition to providing a controlled-release system. It simulates the folklore use of curcumin for wound healing, analgesic, and anti-inflammatory effects in India, as a colloid with standard fats (milk and ghee). SLNs are also reported to offer benefits such as film formation and controlled occlusion resulting in increased skin hydration [25]. The latter is especially useful in the wound healing process. Several attempts have been made to prepare nanoparticles of curcumin, but the reported drug payload with respect to lipids is usually ≤10% *w*/*w* [26,27,28,29,30,31,32,33]. Similarly, the drug content of most of the reported SLN formulations is less than 5 mg/mL [34,35,36,37,38,39] and also involves the use of harmful organic solvents for their preparation and development [40].

It was envisaged in this study to systematically design an SLN formulation with a high drug payload (15% *w*/*w*) with respect to the lipid matrix content, without using any harmful organic solvents such as chloroform, DMSO or ethanol for the dissolution of curcumin, since the complete removal of these organic solvents from the formulation cannot be ensured and they may have toxic implications even when present in small amounts. It was endeavored to develop and optimize the aqueous dispersion of curcumin-loaded solid lipid nanoparticles (CSLNs) using GRAS components, implementing the Taguchi design followed by the central composite design targeted for maximal drug payload, drug content, and encapsulation, and to achieve a controlled release.

However, CSLNs area thin aqueous dispersion which is not suitable for topical application. Furthermore, it has been implicated that wound dehydration disturbs the ideal environment to stimulate the wound healing process; therefore, the maintenance of a moist wound bed is another important consideration for effective wound healing. Hence, CSLNs were incorporated within a three-dimensional polymeric network of a hydrogel. The hydrogel would absorb the tissue exudates, prevent wound dehydration, and allow oxygen to permeate [41].Moreover, the mechanical properties of hydrogels in terms of porosity (allowing the absorption of large volumes of wound exudate), flexibility (adaptation to wound geometry), viscosity, shear stress (deformability adapted to the skin), and elasticity also aid in expediting the healing process, thus establishing a synergism between the CSLNs and their hydrogel cargo.

The optimized CSLN formulation was assessed for its wound healing potential in an in vivo full-thickness excision wound model and a nitrogen mustard gas model. To delineate the mechanism of wound healing, in vitro keratinocyte and fibroblast proliferation effects, in vivo antioxidant (biochemical parameters), in vivo anti-inflammatory and angiogenesis, and in vitro antimicrobial effects of the CSLNs were also assessed. Further, in vitro cytotoxicity and in vivo acute and repeated dose dermal toxicity studies were also conducted to establish the safety of the prepared formulation.

## 2. Materials and Methods

Curcumin extract (95%) was a kind gift from Sunpure Extract Pvt Ltd., Dilshad Garden, Delhi, India; Compritol^®^ 888 ATO was a gift sample from Gattefosse, France; and Phospholipon 90 G (soya lecithin) was gifted by Lipoid, Germany. All the other reagents used in the study were of analytical grade. Mice (wound healing), rats (repeated dose dermal toxicity study) and rabbits (acute dose dermal toxicity) were used for the animal studies.

### 2.1. Development of Curcumin Solid Lipid Nanoparticles (CSLNs)

The CSLNs were prepared by a hot high-pressure homogenization method. The curcumin (0.6% *w*/*w* in the final formulation) was dissolved in polyethylene glycol (PEG) 600 followed by the addition of molten Compritol^®^ 888 ATO. A primary crude emulsion was prepared by emulsifying this hot lipid phase with the aqueous surfactant phase containing Tween 80 and Phospholipon 90G (soya lecithin), maintained at a temperature 5–10 °C above the melting point of Compritol^®^ 888 ATO (70 °C to 75 °C) using a high-speed stirrer (WiseTis HD 15D, Am Bildacker 16, 97877 Wertheim, Germany) at 8000 rpm for 8 min. The coarse emulsion was subjected to high-pressure homogenization (HPH) using the Emulsiflex C3 Avestin (2450, Don Reid Dr. Ottawa, ON, Canada) homogenizer at 1000 bars and three cycles. The dispersion obtained was allowed to cool to room temperature, forming lipid nanoparticles by re-crystallization of the hot dispersed lipid.

### 2.2. Pre-Screening

Various surfactants and co-solvents, such as Tween 80, Gelucire 44/11, glycerol, propylene glycol, PEG300, PEG400, PEG600, PEG4000, PEG6000, Transcutol, and Labrafac, were used to assess the solubility of curcumin. PEG400, PEG600, and Gelucire 44/11, which showed the highest solubility, were used as co-solvents to prepare the different formulations, and Tween 80 was selected as the surfactant of choice.

#### 2.2.1. Preliminary Screening Study

A Taguchi L8 orthogonal array design (trial version 11.0.0; Stat-Ease, Inc., Minneapolis, MN, USA) was used to assess the effect of the parameters viz. concentration of the lipid (Compritol^®^888 ATO; 3 or 5% *w*/*w*), Tween 80 (8 or 12% *w*/*w*), PEG 600 (5 or 8% *w*/*w*) and Phospholipon 90G (0.4 or 1% *w*/*w*), speed of homogenizer (6000 or 10,000 rpm) and homogenization cycles (3 or 6) on the particle size, polydispersity index and entrapment efficiency of the formed CSLNs (Table 1).

#### 2.2.2. Optimization Study

From the preliminary screening test by the Taguchi design, the concentrations of Tween 80 and PEG600 were found to have a significant effect on the particle size, polydispersity index (PDI) and entrapment efficiency of the CSLNs. After selecting important parameters, the response surface methodology using the central composite design (CCD) was utilized to explore the influence of the two factors, i.e., Tween 80 (X1) as the surfactant and PEG600 (X2) as the co-solvent on the formed CSLNs. A CCD comprises of 3 groups of design points, which involves a two-level factorial design, center and axial or star, points resulting in studying the selected factors at 5 different concentrations encoded as α, −1, 0, 1, and +α. The real values of the variables are given in Table 2. A total number of 13 experiments, including 4 factorial points, 4 axial points, and 5 replicates at the center point were performed to estimate the pure error sum of squares. The optimization was performed with the objective of formulating CSLNs with a minimum particle size (Y1), and a maximum entrapment efficiency (Y2). This design was used to select the best-fit model amongst the linear, quadratic and two-factor interaction model due to the analysis of the variance F-value. Estimating the response through the second-order polynomial equation is as shown below:

$$Y_{i}=A_{0}+A_{1}X_{1}+A_{2}X_{2}+A_{3}X_{1}X_{2}+A_{4}X_{1}^{2}+A_{5}X_{2}^{2}$$

where *Y* is the predicted response(s), *A*_0_ is the intercept, *A*_1_ and *A*_2_ are the linear coefficients, *A*_3_ is the interaction coefficient, *A*_4_ and *A*_5_ are the squared coefficients, and *X*_1_ and *X*_2_ are the independent variables. By using this equation, it is possible to evaluate the linear, quadratic, and interactive effects of the independent variables on the responses appropriately. The Design Expert software was employed for the statistical analysis and graph plotting. ANOVA was used to assess the effect of independent variables on the responses, and a *p*-value < 0.05 was considered statistically significant. Multiple correlation coefficient (R^2^) and adjusted R^2^ were used as quality indicators to evaluate the fitness of the second-order polynomial equation. Contour and three-dimensional surface plots assessed the relationship and interaction between the coded variables and the responses. The equation derived from the quadratic model was used to determine the optimal points. The check points comprising five formulations, including the optimized formulation, were prepared to carry out validation. The linear correlation plots between the observed and predicted responses for these formulation check points were constructed and the percent prediction error between the observed and the calculated values were determined to ratify the prognostic ability of the experimental methodology. The selected optimized formulation was used for further studies.

### 2.3. Characterization and CSLN Hydrogel

Determination of the total drug content (TDC)/assay (S1.2.1.1), determination of the entrapment efficiency (EE) (S1.2.1.2), particle size analysis and polydispersity index (S1.2.1.3), zeta potential (S1.2.1.4), preparation of the CSLN hydrogel (S1.2.2), total drug content (drug assay, S1.2.3), and texture analysis of the CSLN hydrogel (S1.2.4) are included in the Supplementary Data.

#### 2.3.1. Field Emission Scanning Electron Microscopy (FESEM)

The CSLNs were observed using an FESEM H-7500 (Hitachi Ltd., Chiyoda, Tokyo, Japan) for determination of the shape, size, and any physical instability, i.e., aggregation or irregularity in the particles. The narrow probing beams of FESEM at low and high energies provide magnified spatial resolution with minimum sample damage. The topographical information is provided at magnifications ranging from 250 to 1,000,000× with ion-free pictures. A thin film of diluted CSLN dispersion was placed on the carbon-coated copper grid and dried before its observation under FESEM.

#### 2.3.2. Transmission Electron Microscopy (TEM)

The CSLNs were suitably diluted (10×) with distilled water for observation. A drop of previously diluted CSLNs was spread to form a film on a copper grid (carbon coated) which was then observed under HRTEM (FE Tecnai G2 F20, Sauletekio av. 3, Vilnius, LT-10257, Lithuania) at a voltage of 200 kV, for evaluation of the size, sphericity, and aggregation.

### 2.4. Stability Studies

The CSLNs were placed in stability chambers at 5 ± 3 °C and 30 ± 2 °C with 65 ± 5% RH as per ICH guidelines for evaluating long-term stability. The samples were withdrawn at months 0, 1, 3, 6, and 12 intervals and evaluated for curcumin assay (total drug content), entrapment efficiency, particle size, and PDI.

#### 2.4.1. Photostability Studies

The photostability studies were conducted as per ICH guidelines on curcumin-loaded SLNs (CSLNs) and free curcumin (curcumin dispersion in 1% CMC). The samples were kept in clear and amber-colored glass containers and charged in the photostability chamber for 10 days at an illumination of NLT 1.2 million lux hours. After 10 days, free curcumin was evaluated for the amount of curcumin left after degradation (assay) whereas the CSLNs were evaluated for total drug content, entrapment efficiency, particle size, and PDI.

#### 2.4.2. Autoclavability

The prepared CSLN formulation was autoclaved at 121 °C for 15 min and observed for any change in the drug assay/total drug content, entrapment efficiency, particle size, PDI, and zeta potential.

### 2.5. In Vitro Studies

#### 2.5.1. Cell Proliferation Assay

Immortal human keratinocytes cell lines (HaCaT; passages 37–39) and human dermal fibroblast cells (FB; passages 2–6; Lifecell Technologies, Aurangabad, India) were grown as described elsewhere [42]. Briefly, Dulbecco’s modified Eagle medium (DMEM; Life Technologies, Carlsbad, CA, USA) including 10% *v*/*v* fetal bovine serum (FBS; Life Technologies) and 1% *v*/*v* penicillin–streptomycin (P/S; Sigma Aldrich, Darmstadt, Germany) was used for the cell culture. After 80–90% of cell confluency, the cells were trypsinized and counted for this experiment. The cells (5000 for HaCaT; 2000 for FB) were plated in a 96-well plate overnight in regular media and then washed with 1× PBS (twice) and switched to the serum-free media, including different conditions such as a control (serum-free media only), blank SLNs (BSLN), free curcumin dispersion (FC), and CSLN (0.01–8 µg/mL in serum-free media), and incubated for the next 48 h. After this, the media were replaced with fresh media containing 10% Alamar Blue reagent (Alamar Blue; Life Technologies, Carlsbad, CA, USA) according to the manufacturer’s instructions, and incubated for 1 h. The fluorescence of the wells was read after 1 h (DTX 880 Multimode Detector, Beckman Coulter, Fullerton, CA, USA) and the data were compared between the groups.

#### 2.5.2. In Vitro Release Studies

Jacketed Franz diffusion cells were used to assess the in vitro release of free curcumin and curcumin from the CSLNs. These cells consist of the donor and the receptor chamber between which the dialysis membrane is positioned. The dialysis membrane was soaked in double-distilled water for 12 h before use. The whole system was water-jacketed and thermostatically controlled by an external circulating water bath at 37 ± 1 °C. The receptor media consisted of 50% methanol in distilled water. Initially, a pH 6.8 buffer was used as the receptor medium, but it resulted in simultaneous degradation of curcumin so that estimations could not be made correctly. The CSLN and the corresponding concentration of free curcumin (0.5 mL each) dispersed in 1.0% (*w/v*) CMC was poured evenly on the donor side covered with a paraffin film to avoid evaporation of the loaded sample. The receptor media were stirred throughout the experiment, using a magnetic stirrer. The entire cell was covered with aluminum foil to prevent photodegradation of the curcumin. Aliquots (1 mL) were withdrawn regularly from the receptor compartment at various times, starting at 30 min, and replaced by an equal volume of fresh receptor medium. The samples were analyzed immediately, spectrophotometrically, at 425 nm after a suitable dilution when required. The safety studies are included in the Supplementary Data (S1.2.5).

### 2.6. In Vivo Wound Healing Activity

#### 2.6.1. Nitrogen Mustard (NM)-Induced Burn Wound

Female CD-1 mice were used for the present study. They were housed under a 12-h light/dark cycle and were allowed to acclimatize for at least one week before the study. They were fed a standard diet and water ad libitum. All the animal studies were performed in the Association for Assessment and Accreditation of Laboratory Animal Care International-accredited animal facilities, under approved protocols from the Rutgers University Animal Use and Care Committee.

The mice divided into four groups (6 animals/group), a nitrogen mustard (NM) control (only nitrogen mustard), CSLN hydrogel (NM followed by CSLN treatment twice a day containing 50.6 μg curcumin per application), a naïve control (no NM, no drug, no vehicle) and a vehicle control (Pluronic F127-based thermo-sensitive gel with d-α-tocopheryl polyethylene glycol 1000 succinate treatment; no NM), were anesthetized via isoflurane under the fume hood. Acetone (10 μL) was used to adhere 6 mm glass microfiber filter discs (GE Healthcare, Buckinghamshire, UK) on either side of the shaved skin of the lumbar region of the mice, at equal distance from the spine. The freshly prepared 1M NM HCl (20 μL; 20 μmol) in 20% deionized water/80% acetone *v*/*v* was then applied to the filters, which were then immediately covered with PARAFILM^®^ (Pechiney, Menasha, WI, USA). The PARAFILM^®^ and filter discs were removed after 6 min and the mice kept in the fume hood, housed 2 per filter top cage with a removable divider separating them, were then allowed to recover from anesthesia. The CSLN/vehicle hydrogel was gently rubbed into the skin of the wounded areas, three hours post NM application. The treatments were repeated on days 2 and 3. The mice were relocated from the fume hood to the vivarium on the 2nd day. On the fourth day, 12 mm, full-thickness punch biopsies of the exposure site and surrounding tissue were collected using a biopsy punch. The biopsies were weighed (mg) with an analytical balance to assess the degree of skin inflammation relative to the control groups [43].

#### 2.6.2. Full-Thickness Excisional Wound

Lacca mice were used for the study and were divided into six groups (*n* = 8). The animals were depilated on the paravertebral area before wound creation and a circular excision wound of 6 mm in diameter was created using a biopsy punch. The animals were treated with a free curcumin Carbopol 934P hydrogel (200 mg containing 1 mg of curcumin), a blank SLN Carbopol 934P gel, marketed formulation (Soframycin^®^) and a CSLN Carbopol 934P gel (200 mg of hydrogel containing 1 mg of curcumin) once daily for 11 days. Two control groups (naïve control and positive control (full excision wound but no treatment)) were also included in the study. The animals with undressed wounds were housed individually. The progressive reduction in the wound area of the animals, expressed as mm^2^, was monitored planimetrically by tracing the wound margin on a graph paper every alternate day for 11 days.

#### 2.6.3. Effect of Topical Application of CSLNs on Lipid Peroxidation, Reduced Glutathione and Antioxidant Enzyme Catalase

A 10% *w*/*v* wound tissue homogenate was prepared in cold 10% *w*/*v* potassium chloride and centrifuged at 10,000× *g* for 15 min. The oxidant/antioxidant status of the wound tissue was determined on day 11 by quantifying the levels of lipid peroxidation (LPO) [44], reduced glutathione (GSH) [45], and catalase [46] in the prepared homogenates.

#### 2.6.4. Effect of Topical Application of CSLNs on TNF-α and VEGF

The TNF-α and VEGF levels in the wound tissue homogenate were determined by ELISA as per protocols given in the Murine TNF-α Mini ABTS ELISA Development Kit (Catalog#900-M54, Lot# 0916054-M) and the Murine VEGF Mini ABTS ELISA Development Kit (Catalog# 900-M99, Lot# 0812099-M), respectively.

#### 2.6.5. Histology and Immunohistochemistry

At the end of the study the mice were sacrificed, and the skin tissue was excised including the wound scar area, and then processed for histological studies as previously described [47,48]. Briefly, tissues were fixed initially in 10% formalin (48–72 h shaking) and then stored in 70% ethanol at 4 °C until processed. The tissues were then paraffin-embedded and 5 µm-thin sections were cut from the middle of the wound and stained with hematoxylin and eosin (H&E) to visualize the tissue morphology and measure the thickness of the epidermis and dermis using ImageJ (NIH). For immunohistochemistry, the tissues were de-paraffinized and rehydrated, followed by antigen retrieval (2100 Antigen Retriever). Sections were then rinsed in PBS, endogenous H_2_O_2_ blocking (3% in PBS, 20 min), nonspecific blocking with a serum-free protein (20 min at RT; Dako, Denmark), and endogenous biotin-blocking using an avidin/biotin reagents kit (Vector Laboratories, Burlingame, CA, USA), including washing sections (5 min × 2) at each step by Tris-buffered saline with Tween-20 (TBST). Then, the sections were stained overnight with the primary antibodies, namely, anti-alpha-smooth muscle actin (a-SMA; rabbit monoclonal IgG, 1:2000; Epitomics, Inc., Burlingame, CA, USA), CD31 (rabbit polyclonal IgG, 1:200; Abcam, Cambridge, MA, USA), and Ki67 proliferation antigen (rabbit monoclonal IgG, 1:1000; Lab Vision Corporation, Fremont, CA, USA) followed by a secondary antibody (biotinylated goat anti-rabbit, 1:200; Vector Laboratories). The sections were then washed (TBST, 5 min, twice), stained with working ABC reagent (Vectastain ABC Kit, 30 min at RT; Vector Laboratories), NovaRed solution (5 min at RT), and counterstained with Mayer’s hematoxylin and Scott’s bluing reagents, with intermediate washes with dH_2_O. The sections were then dehydrated and mounted using Cytoseal 60. The sections were also stained with normal rabbit Ig fraction (1:400,000; Dako, Santa Clara, CA, USA) overnight at 4 °C as a negative control. The positive cells/area (sum of dermis and epidermis) for CD31, Ki67, and a-SMA, in the stained sections, were determined by ImageJ (NIH, Bethesda, MD, USA). Two sections were averaged for each animal (*n* = 2).

### 2.7. Antimicrobial Activity of CSLNs

#### 2.7.1. Effect against Planktonic Cells

The effect of CSLNs, blank SLNs, and free curcumin (FC) dispersed in 1% *w*/*v* CMC on the planktonic growth of *Staphylococcus aureus 9144* was evaluated. Varying (16 to 2048 µg/mL of curcumin) concentrations of CSLNs, blank SLNs, and FC, prepared in Mueller–Hinton broth No. 2 control cations (MH) medium was used to dilute 6.5 µL of 5 × 10^7^ CFU/mL of *Staphylococcus aureus 9144* cell suspension to 650 µL. Each dilution (200 µL) containing 5 × 10^5^ CFU/mL was added to a 96-well plate and incubated at 37 °C for 24 h. The wells without any test substance served as controls since the color of curcumin and the opaqueness of the SLN formulation at high concentrations can interfere with the absorbance readings, therefore the plate count method, though a lengthy and more cumbersome process, was preferred over the optical density measurement to calculate the MIC. The lowest concentration of each test material that caused ≥50% reduction in the cells in comparison with the control was noted as the MIC.

#### 2.7.2. Effect against Biofilm Formation

The biofilms of *Staphylococcus aureus 9144* were developed in polystyrene microplates using an in vitro biofilm method [49]. Varying (i.e., 16 to 2048 µg/mL) concentrations of CSLNs, blank SLNs, and FC were prepared in brain heart infusion (BHI) broth. *Staphylococcus aureus 9144* cell suspension (13 µL of 1.5 × 10^8^ CFU/mL) was diluted to 650 µL with the previously prepared concentrations of CSLNs, blank SLNs, and FC. Each dilution (200 µL) containing 3 × 10^6^ CFU/mL was added to a 96-well plate and incubated at 37 °C for 24 h.

#### 2.7.3. Effect against Mature Biofilms

The effect on mature biofilms was studied by initially incubating only the BHI broth containing 3 × 10^6^ CFU/mL of *Staphylococcus aureus* 9144 at 37 °C for 24 h, followed by washing with PBS to remove the planktonic cells. After washing, varying (i.e., 16 to 2048 µg/mL) concentrations of CSLNs, blank SLNs, and FC prepared in the BHI broth were added to a 96-well plate which was then incubated at 37 °C for 24 h.

Biofilm growth was analyzed using the XTT metabolic assay. The XTT solution of 1 mg/mL concentration was prepared in PBS and stored at −20 °C until use. The menadione solution, prepared in acetone, was added to the XTT solution to get a concentration of 4 μM. The wells containing biofilms were washed with PBS, and 100 μL of the XTT–menadione solution was added to each, followed by incubation at 37 °C for 5 h in the dark. The intensity of the color formation by the water-soluble formazan product was measured at 450 nm using a microplate reader, which indicated the metabolic activity relative to the biofilm growth (i.e., relative metabolic activity (RMA)). The wells without the test compounds were considered as the control, while the wells without biofilms served as the blanks. The lowest concentration of the test molecule causing ≥50% reduction in the RMA was considered the MIC for the biofilm.

## 3. Statistical Analysis

All data were analyzed statistically using ANOVA in Prism 6.01 GraphPad Software, 2365 Northside Dr., Suite 560, San Diego, CA 92108. The *p*-values considered for significance are indicated for respective figures.

## 4. Results and Discussion

### 4.1. Pre-Screening

Curcumin was not found to be soluble in any of the lipids evaluated for the study. These included stearic acid, glyceryl monostearate, Compritol^®^ ATO 888 and cetyl alcohol. Compritol^®^ ATO 888 was, however, chosen as the lipid based on our prior experience and literature reports, which show that it results in stable, safe, uniformly sized, and spherical particles which encapsulate significant amounts of hydrophobic drugs [39,49,50]. Various surfactants were evaluated for their property to dissolve curcumin and also to increase its solubility in Compritol^®^ ATO 888. Amongst a list of surfactants tested, Tween 80 was found to show the best results. Further, curcumin was found to be highly soluble in polyethylene glycol (PEG) 400, PEG 600 and Gelucire 14/44. Hence, they were taken as cosolvents to dissolve curcumin in the lipidic phase whereas Tween 80 was selected as a surfactant for the aqueous phase. Various formulations were then prepared by varying the concentration of Tween 80 and the cosolvents (Table 3). The formulations were observed for stability (no separation of phases), drug expulsion and crystallization. Based on pre-screening, Tween 80 and PEG 600 were selected as the surfactant and cosolvent of choice for screening in the optimization studies. PEG is a non-ionic glycol and acts as a solubilizer, plasticizer and permeation enhancer. The oxygen atoms present in PEG give it hydrophilicity while the CH_2_–CH_2_ groups display lipophilicity [51]; and it seemed to have a special role in ensuring the effective encapsulation of curcumin in the SLNs and in keeping any unencapsulated drug in the solution. F12 did not show any drug crystals but the total concentration of surfactant used was very high (26.9%). So, an attempt was made to decrease the overall surfactant concentration by increasing the concentration of Tween 80 to 12%, and further formulations were prepared (F13–F16) and observed for stability in terms of the separation of phases or drug expulsion and crystallization when observed under a microscope, and F16 was selected for further optimization studies.

### 4.2. Screening Studies

**Figure 1:**
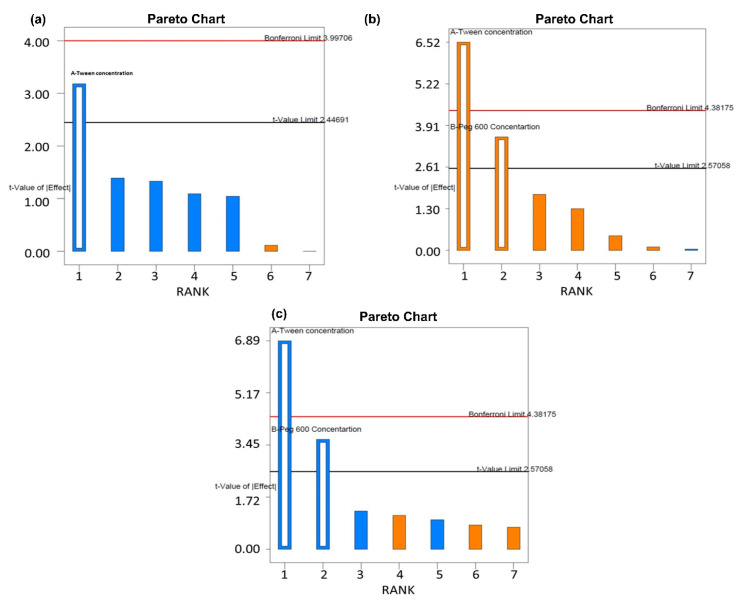
Pareto charts for response variable (**a**) particle size, (**b**) % entrapment efficiency, and (**c**) PDI.

The most significant product or process variables that affect the quality performance of CSLNs were screened using the Taguchi orthogonal array design. The advantages of this design include the least number of runs (i.e., 8) and an expansive number of variables (up to 7) that can be evaluated. Table 1 enlists various factors and responses as per the Taguchi design. The numerical model generated for each response variable was found to be significant (*p* < 0.005). The pareto charts of particle size, EE % and PDI were used to assess the effect of various factors on the response variables, as represented by Figure 1. The most significant factors as per the pareto charts were the respective concentration of Tween 80 and PEG 600. Hence, they were selected for further evaluation using the CCD design.

### 4.3. Statistical Analysis of Experimental Data by Design Expert Software

After preliminary optimization (Table 1), the concentrations of Tween 80 and PEG 600 were selected as independent variables based on their effect on response parameters, particle size (Y1) and entrapment efficiency (Y2). The CCD was used to conduct thirteen experimental runs whose design layout and values of formulation parameters are given in Table 2. All the batches were optimized on the basis of Y1 and Y2. The value of responses Y1 and Y2 ranged from 150.3 to 598.6 nm and 45.9 to 76.8%, respectively. According to the ANOVA results calculated by Design Expert software, quadratic second-order polynomial was the best model for data fitting (Table 4). The selection of a model for analyzing the responses was done based on lack of fit (LOF) and model summary statistics. The LOF indicates the adequacy of a model for fitting the experimental data, whereas the non-significant value of LOF signifies the suitability of the model to fit the experimental data. As can be seen, the ANOVA F-value (357.71 and 34.54 for Y1 and Y2, respectively) of the model for both the responses indicated that the model *p*-value is less than 0.05 and is thus significant, while LOF is not significant (F = 4.09 and 4.27 for Y1 and Y2, respectively; *p* > 0.05) confirming its suitability for the prediction of the responses and finding the optimum conditions.

The suitability of the polynomial model equation for particle size (R^2^ = 0.9961) and % EE (R^2^ = 0.9610) fitting was expressed by the respective coefficients of determination. The latter indicated that the model could explain 99% and 96% variability, respectively, in the responses Y1 and Y2, and only 1% and 4% of the variability is due to noise, respectively. The predicted R-squared (Pred R^2^) values for the responses (Y1 = 0.9775 and Y2 = 0.7754) were found to be in reasonable agreement with the adjusted R-square values (Adjs R^2^) (Y1 = 0.9933 and Y2 = 0.9332) indicating that the model predicted the responses accurately.

The standard deviation associated with the experiment around the mean can be evaluated from the value of the coefficient of variation (CV% = 3.69 and 4.07 for Y1 and Y2, respectively). Further, the CV (%) signifies the precision and reliability of the model used. Finally, the predicted R^2^ of 0.82 implies the good prediction of the particle size by the model. Adeq. Precisions for the responses Y1 and Y2 were 55.512 and 18.634, respectively (Table 4). Adeq. precision measures the signal-to-noise ratio and a ratio greater than 4 is desirable.

#### 4.3.1. Effect on Particle Size

The regression equation for the particle size in terms of coded factors is derived as:

$$Y_{1}=+3969.53753-443.0278*X_{1}-303.97911X_{2}+14.775X_{1}X_{2}+13.90844X_{1}^{2}+8.17056\;X_{2}^{2}$$

**Figure 2:**
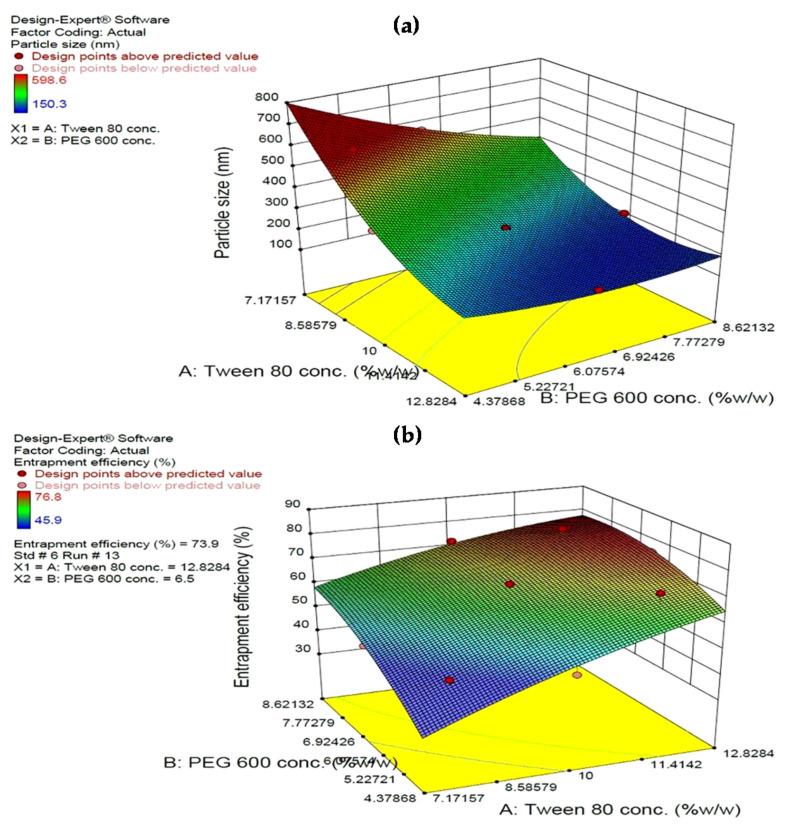
Response surface curves for (**a**) particle size, and (**b**) entrapment efficiency.

**Figure 3:**
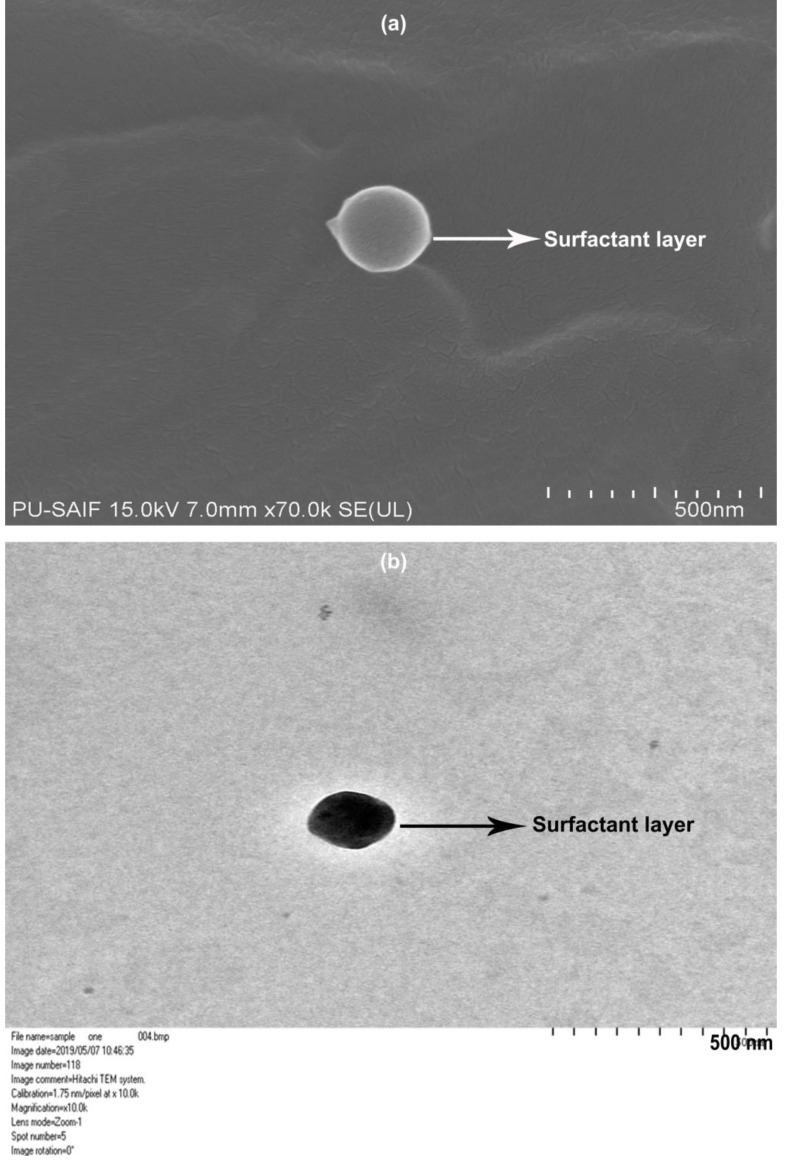
(**a**) FESEM image of the CSLNs (70,000×), (**b**) TEM image of the CSLNs (10,000×).

Generally, in the coded equation higher positive coefficients indicate that increasing the variable value will increase the response, and vice versa, and confirms that a lower amount of variable is desired. The magnitudes of the coefficients indicate the relative contributions of the factors into the response. Quantitative estimation of the significant models indicated that both Tween 80 and PEG 600 have prime influence on the particle size for their large negative coefficient, suggesting that increasing the amount of both Tween 80 and PEG 600 in the formulation decreased the particle size. It was observed that the prevalence of an acceptable blue region was quite low at lower levels of Tween 80 and the acceptable blue region increased with the gradient inclination of Tween 80 indicating the significant reduction in size. A similar trend was observed with PEG 600 also but the effect observed with Tween 80 was more pronounced. It may be concluded that keeping the amount of lipid constant, increasing the concentration of surfactant, resulted in a decrease in particle size. During the homogenization process, the high shearing forces reduced the particle size. However, on removing the applied shear forces, the smaller droplets tended to aggregate to attain a low surface energy [52]. The kinetics of surfactant adsorption on newly created hydrophobic surfaces of SLNs is mainly diffusion controlled. In this case, the surfactant concentration in the aqueous phase becomes an important driving force. At excessively low surfactant concentration, the surfactant molecules are either insufficient to completely cover the newly created particle surface, resulting in suboptimal stabilization of the particle dispersion, or they are not adsorbed at a rate rapid enough to match or supersede the rate of particle–particle collisions resulting in particle aggregation. However, the molecular characteristics of the surfactant and the nature of the interface also influence the kinetics of the adsorption process. Moreover, the amount of surfactant required to maintain the stability of the hot SLN dispersion during the cooling/crystallization process depends on the relative surface area increase [53]. The presence of the surfactant molecule as a thick protective covering around the solid lipid droplet in water emulsion is observed presently in the FESEM and TEM images (Figure 3a,b shown). The former can effectively avoid aggregation. The interaction patterns between the variables were studied by three-dimensional response surface curves. The latter were graphed by estimating responses Y1 and Y2 at various levels of X1 and X2, as shown in Figure 2. The curvature along the X1 axis reveals the statistical significance of the quadratic coefficient of the concentration of Tween 80 in the model.

#### 4.3.2. Effect on Entrapment Efficiency

The quadratic polynomial equation of the %EE in terms of coded factors was determined and is presented in equation as below:

$$Y_{2}=-130.16712+15.55617*X_{1}+24.52045X_{2}-0.59167X_{1}X_{2}-0.35281X_{1}^{2}-1.071672X_{2}^{2}$$

EE was found to increase with the increase in the concentration of both Tween 80 and PEG 600. A higher coefficient for the latter indicates its higher influence on EE, which could be attributed to the increased solubility of curcumin in the lipid core by the solvent action of PEG 600. Further, PEG 600 being highly hydrophilic has a high affinity for the aqueous phase. Therefore, in addition to improving solubility of curcumin in the lipidic phase, PEG 600 having a high affinity for water will move towards the aqueous phase, in turn leaving behind/precipitating curcumin in the lipidic core. Curcumin, being hydrophobic, does not diffuse to the aqueous phase thereby improving both its payload and entrapment efficiency. Furthermore, the surface coverage of nanoparticles with PEG 600, which is both a surfactant-supporting agent and a plasticizer [54], prevents drug leaching from the lipid matrix [55]. An increase in the surfactant concentration increases the entrapment efficiency of curcumin within the SLNs [38]. In Figure 2, the response surface plot shows the impact of X1 and X2 on % EE.

#### 4.3.3. Optimized Formulation and its Validation

The composition of the optimum formulation was determined as 12% (*w*/*v*) Tween 80 and 8% (*w*/*w*) PEG 600, which fulfilled the requirements of optimization for minimum particle size and maximum entrapment efficiency. At these levels, the predicted values of Y1 and Y2 were 156.6 nm and 76.5%, respectively. The check point validation of the predicted values showed close observed values with % error in all cases being <±6.33 (Table 5).

### 4.4. Characterization

#### 4.4.1. Assay/TDC and EE

The total drug content of 5.8 ± 0.2 mg/mL was observed, amounting to 96% of the original amount incorporated (6 mg/mL), confirming that insignificant losses occurred during the process of preparation of the SLNs by high-pressure homogenization (*n* = 6). An EE of 75.55 ± 2.31% was observed (*n* = 12). We previously [39] reported a micro-emulsification method for the preparation of curcumin-loaded solid lipid nanoparticles with an entrapment efficiency of 81.92 ± 2.91%. The latter, although higher than the presently obtained value, is for a lower drug payload of 10% with respect to the lipid matrix (presently it is 15%) and employed a much higher (>2 times) Tween 80 concentration of approximately 25%. Furthermore, the microemulsion process is a less industry-amenable method. Similarly, a high entrapment efficiency of 84.6% is also reported elsewhere [31], but again at a much lower drug payload of 6.6% to 10% and employing high shear homogenization followed by ultrasonication. The particle size of the SLNs prepared by these workers was 401.9 nm.

#### 4.4.2. Particle Size Analysis, Polydispersity Index, and Zeta Potential

An average particle size of 170.1 ± 26.6 nm and a significantly low PDI of 0.143 ± 0.026 was observed (*n* = 10; Figure S1a in Supplementary Data). A low PDI indicates a uniform distribution of particles around the mean. The zeta potential was found to be −9.67 ± 1.47 (*n* = 6) (Figure S1b in Supplementary Data). Although a zeta potential of >±25 is considered a measure of good stability; however, the present SLN dispersion was also observed to be stable. It is often contested that zeta potential is measured after dilution of the original dispersion and thus does not give a true measure of the charge on the particles. Further, as per the DLVO theory, the colloidal stability is a sum of the van der Waals attractive and electrostatic repulsive forces due to the electron double layer [56]. While zeta potential is a measure of the electrostatic repulsive forces, it does not consider the attractive van der Waals forces [57]. Considering that PEG forms hydrogen bonds with water molecules, we expect some stability being assigned because of that too. Further, a surfactant layer, as observed presently and shown below in the FESEM and TEM studies (Figure 3a,b) around the solid lipid nanoparticle, also attributes to its stability to aggregation.

#### 4.4.3. FESEM and TEM Studies

The FESEM of the CSLN (Figure 3a) formulation showed small spherical particles as individual entities and an absence of any agglomerates indicating their stability. A coat of surfactant on the outside of SLN covering the entire surface of the particle uniformly and clearly visible in the Figure 3a, assigns it with the stability to aggregate. Morphological analysis of CSLN in aqueous dispersions, detected by TEM analysis, also indicates near-spherical particles (spindle shaped) (Figure 3b). The polymorphic form of the SLNs also reflects in the shape of the particles; particles in α modification are usually observed as circular to polyhedral [58]. The data of PXRD (S2.1.1), DSC (S2.1.2), and FTIR (S2.1.3) are included in Supplementary Data.

### 4.5. Stability Studies

An insignificant change in TDC, EE, particle size and PDI was observed when the CSLNs were stored at 5 ± 3 °C for a period of 12 months (Table 6). This may be attributed to the maintenance of the SLNs in their native state, in which they were produced, comprising a suitable concentration of surfactants in the aqueous phase such that they remain dispersed as single particles even during long-term storage. Further, the presence of free/unencapsulated drug in the aqueous compartment maintains a dynamic equilibrium with the entrapped drug so that % EE is uniform during storage. Upon storage at 30± 2 °C and 65% RH ± 5% RH, the change in assay/TDC or EE was <5% after 6 months and it was 10% at 12 months. However, a significant increase in particle size was observed at this temperature. This was manifested as four times increase in the viscosity of the CSLNs; similar results are reported by others [59]. It is reported that an introduction of energy to the SLN system (temperature, light) leads to particle growth and subsequent gelation. It has been postulated that a high film rigidity of the emulsifier (microviscosity) avoids fusion of the film layers after particle contact. However, this microviscosity is a temperature-dependent factor. Hence, an increase in temperature (from 5 to 30 °C) results in a decrease in microviscosity, resulting in destabilization and aggregation of the particles [60]. The evaporation of water could also be responsible for the increased viscosity and subsequent particle size as stability studies were conducted in polyethylene terephthalate bottles which are not air-tight or water vapor resistant.

#### 4.5.1. Photostability

Photostability studies were performed to establish the shielding effect of SLNs lipid matrix offered to curcumin (Table 7). From the results, it is obvious that the CSLNs are completely protected in contrast to the free curcumin which underwent significant degradation. Free curcumin in solid or dissolved form absorbs strongly in the visible wavelength range, making it predisposed to degradation and modification in daylight and artificial lighting [61,62]. The SLNs stored in amber-colored and transparent containers did not show any degradation in terms of assay/TDC and entrapment (*p* > 0.05). The PDI was also similar; however, some increase in particle size was observed (Table 7). On the other hand, 21.7% and 35% of the free curcumin was found to be degraded upon storage in amber-colored containers and transparent containers, respectively.

#### 4.5.2. Autoclavability

The developed CSLNs were found to be stable to autoclaving with no change in entrapment efficiency. A slight increase in particle size was observed, as shown in Table 8. Similar observations are also reported by others [63]. This could be attributed to the high temperature reached during sterilization by autoclaving, which results in the formation of hot o/w microemulsion in the autoclave and probably modifies the size of the hot nanodroplets. On subsequent slow cooling, the SLNs are reformed but some nanodroplets may coalesce during cooling, producing larger SLNs than the initial ones [63]. Sterilization is an important requirement for wound dressings especially if they are required to be used on the burn or infected wounds.

Special care has been taken to choose an appropriate sterilization technique to ensure no degradation or aggregation of the solid lipids to avoid toxicity and instability during the sterility of the CSLNs [64]. Further, it should not change the formulation properties including the physical and chemical stability and the drug release kinetics [64]. One of the advantages of SLNs over other colloidal systems is that they can be sterilized by autoclaving, a commonly used and reliable technique. SLNs melt and recrystallize in a controlled manner at high temperatures [65].

### 4.6. In Vitro Studies

#### 4.6.1. Cell Proliferation Assay

**Figure 4:**
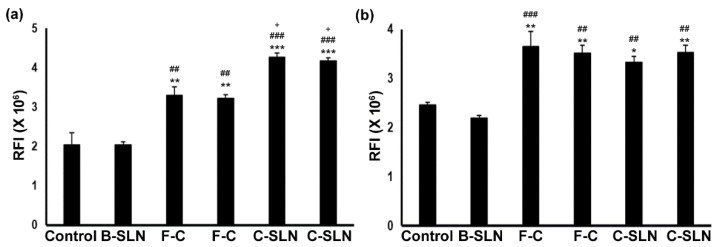
(**a**) Proliferative effect of CSLN on human keratinocytes (HaCaT) and (**b**) fibroblast (FB) cells. The bar heights represent the relative fluorescence intensities (RFI) of reduced Alamar Blue reagent (1 h incubation) after 48 h of treatment with the control, BSLN (8 µg/mL), FC, and CSLN (0.01 and 0.1 µg/mL, respectively). Data are expressed as mean ± SEM (*n* = 3 independent experiments, 9 wells/condition). * indicates a difference between the control to the other groups; # indicates a difference between BSLN to other groups, while + indicates a difference between FC and CSLN. + *p*< 0.05, **/## *p* < 0.01, and ***/### *p* < 0.001.

In these studies, both the cell types respond significantly to the treatments (ANOVA, F = 33.39; *p* < 0.0001 for HaCaT and F = 14.079; *p* = 0.0001 for FB) depending on the concentration (Figure 4a,b) of different experimental conditions. The cells did not show any effect with the blank SLNS (BSLN) treatment up to 8 µg/mL, but both the free curcumin dispersion (FC) (*p* < 0.001) and the CSLN (*p* < 0.001) significantly induced cell growth in HaCaT and FB up to a 0.1 µg/mL concentration, as compared to the control and BSLN. The CSLN treatment induced more growth compared to the FC in HaCaT (Figure 4a, *p* < 0.05). The concentration of over 2 µg/mL of FC and CSLN, however, caused cell death in these experiments (data not shown).

The data of the total drug content/assay of the CSLN hydrogel (S2.2) and texture analysis of the CSLN hydrogel (S2.3) are included in Supplementary Data.

#### 4.6.2. In Vitro Drug Release

**Figure 5:**
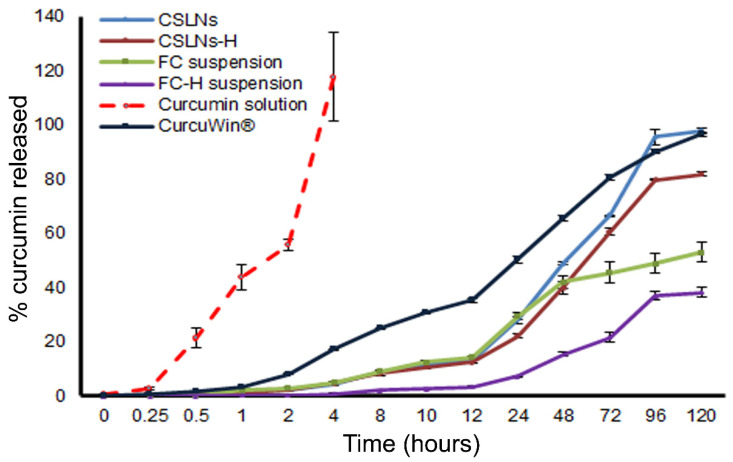
Cumulative % curcumin release from various formulations. CSLN-H—CSLNs hydrogel; FC—Free curcumin; FC-H- Free curcumin hydrogel.

The drug release from the CSLNs and free curcumin is shown below in Figure 5. The release from the CSLNs, CurcuWin^®^ (marketed formulation of curcumin which comprises the dried curcumin powder mixed with hydrophilic polymers such as hydroxylpropyl methylcellulose (HPMC) and polyvinyl pyrrolidine (PVP) claiming its high bioavailability of 136.3%) and the CSLN hydrogel was extended up to 120 h with 98.0 ± 1.9%, 96.63 ± 0.80% and 81.95 ± 0.64% release, respectively. The CSLNs and CSLN hydrogel followed zero-order release kinetics, i.e., controlled release (Table 9). A zero-order model describes the release of drugs/active at a constant rate, which is considered as the optimal kinetics among all controlled-release models [66]. We have previously [39] reported a first-order release for curcumin-loaded solid lipid nanoparticles prepared by the micro emulsification technique. In another study [67], the biopolymeric double-layered SLNs release followed the Higuchi and Weibull model, and the authors reported a zero-order release when the double layer around these SLNs was crosslinked, ensuring a slow diffusion of curcumin.

No burst release was observed in the current study in the cases of the CSLNs as well as the CSLN hydrogel, indicating that curcumin was homogenously dispersed within the lipidic core and was not adsorbed on the surface, resulting in its slow release in a controlled manner. This could be attributed to the inclusion of PEG 600 in the SLN composition, which achieves complete solubilization/encapsulation of curcumin in the lipidic phase. Further, a high affinity of PEG 600 for the aqueous phase prevents the diffusion of curcumin into the aqueous phase during the addition of the lipidic phase into the aqueous phase for SLN production. PEG 600 itself moves into the aqueous phase leaving behind/precipitating curcumin in the lipidic core. Furthermore, a layer of surfactant on the SLN surface, as observed in the FESEM and TEM images, also contributes to the slow diffusion of curcumin from the interior of the SLNs. Hence, the sustained release was observed in the case of CSLNs, whereas a burst release was observed with CurcuWin^®^. A first-order release was observed as expected with free curcumin solution in methanol and the drug was completely released within 8 h whereas only 53% and 38.23 ± 1.78%, respectively, of curcumin was released from the free curcumin suspension and the free curcumin suspension hydrogel in 120 h. The latter also showed zero-order kinetics but incomplete (only 38%) release even after 5 days.

In consideration to the poor aqueous solubility and the instability of curcumin at various physiological pH, the selection of an appropriate release media was of utmost importance. Due to the significant solubility of curcumin in methanol, different proportions were mixed with solutions buffered to pH 6.8 (skin) and pH 7.4 (plasma). However, the mixing of methanol with buffers led to the precipitation of electrolytes from the solution system. The latter was resolved by replacing methanol with ethanol in the buffered system, but the latter was found to degrade the released curcumin. Hence, methanol:water (1:1) was chosen as the satisfactory release medium. Varying concentrations of methanol ranging from 10 to 50% were tried; however, only the 50% methanol in the water combination could solubilize enough curcumin to maintain a sink condition.

### 4.7. Safety Studies

The safety studies are presented in Supplementary Data (S2.4).

### 4.8. In Vivo Wound Healing Studies

#### 4.8.1. Nitrogen Mustard-Induced Burn Wound and CSLNs

**Figure 6:**
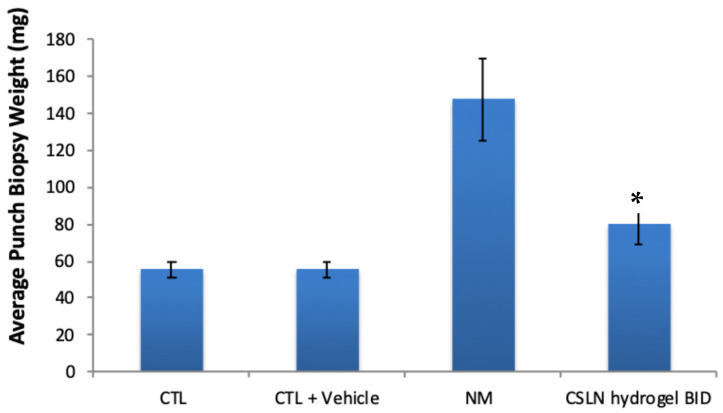
Average punch biopsy weight for different groups, three days post nitrogen mustard exposure. CTL = naïve control, CTL+vehicle = only vehicle applied on control skin; NM = nitrogen mustard treatment; and BID = twice a day. * Significant decrease in inflammation in comparison to nitrogen mustard group.

A significant reduction (184%) in inflammation represented by the average punch biopsy weight was observed in the groups treated with CSLNs in comparison to NM, confirming the anti-inflammatory activity of CSLNs on burn wounds (Figure 6). Curcumin’s anti-inflammatory properties have been unequivocally established [68,69] in organs, including the liver and skin, through the modulation of autoimmune disease and the prevention of injury to these organ tissues [68]. The primary mechanism by which curcumin modulates inflammation is by reducing the expression of the two main cytokines that are released by monocytes and macrophages [24,70]. These molecules are interleukin 1 (IL-1) and TNF-α, which have important roles in the regulation of the inflammatory response. The influence of CSLNs on TNF-α in wound healing is established below in the section on the excision wound. Curcumin inhibits the activity of the proinflammatory transcriptional factor, NF-κB, which is responsible for the regulation of several genes involved in the initial onset of the inflammatory response. Similar anti-inflammatory activity of curcumin has already been reported by us [68], where the application of curcumin vesicular ointment resulted in the suppression of acute inflammation as well as significant inhibition of cotton pellet-induced chronic inflammation.

#### 4.8.2. Full-Thickness Excision Wound Healing and CSLNs

**Figure 7:**
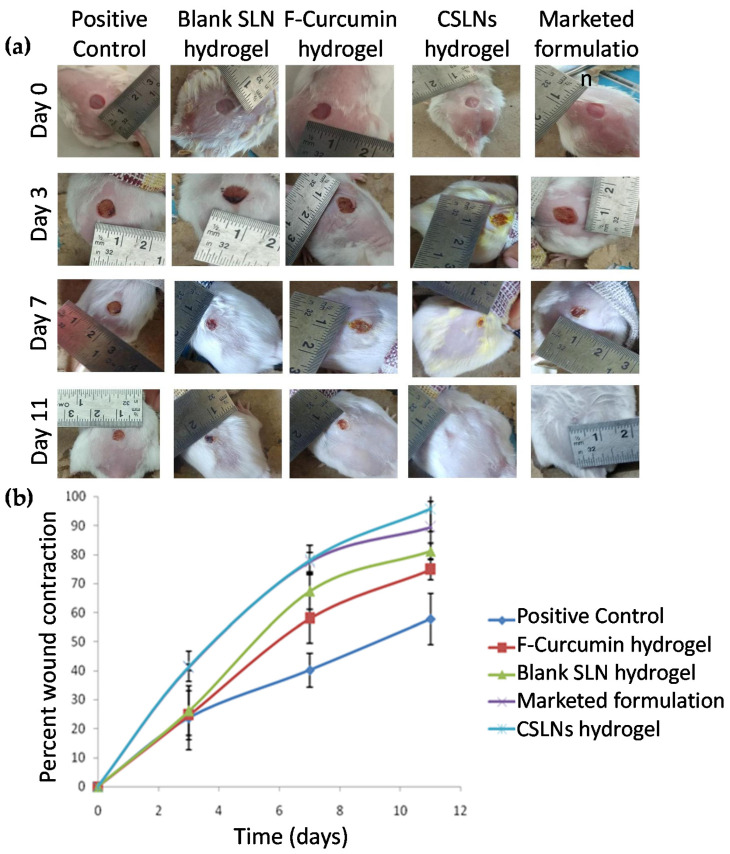
(**a**) Representative wound images taken at day 0, day 3, day 7, and day 11 in different groups, (**b**) percent wound contraction versus days for various treatment groups on different days. F-free.

On day 11, CSLNs resulted in the complete closure of the wounds (95.76 ± 7.85% reduction in wound area) which was significantly better than the positive control (57.85 ± 8.85%), blank SLN hydrogel (81.18 ± 2.66%) and free curcumin hydrogel (74.75 ± 3.53%) (Figure 7a,b). The wound closure was also better than the marketed formulation which resulted in 89.47 ± 8.93% reduction. Interestingly, the blank SLN gel also always showed significant healing comparable to the free curcumin gel (*p* < 0.05) and to the marketed formulation at day 7 and 11 (*p* < 0.05). This could be due to (i) the occlusive nature of SLNs which help to keep the wound moist, (ii) the film-forming nature of SLNs which may protect against the onslaught of pathogenic organisms, and (iii) the presence of 0.4 *w*/*w*% phosphatidyl choline in the SLN composition that has established antioxidant (1–10 mg/mL) and wound healing effects [71]. Oxidative stress is implicated as a deterrent to the wound healing process [72]. Though present in low quantities, its presence as the outer bilayer of the SLNs may improve its efficacy due to a higher surface area and nano nature.

#### 4.8.3. Effect of Topical CSLN Hydrogel Application on Antioxidant Enzymes

**Figure 8:**
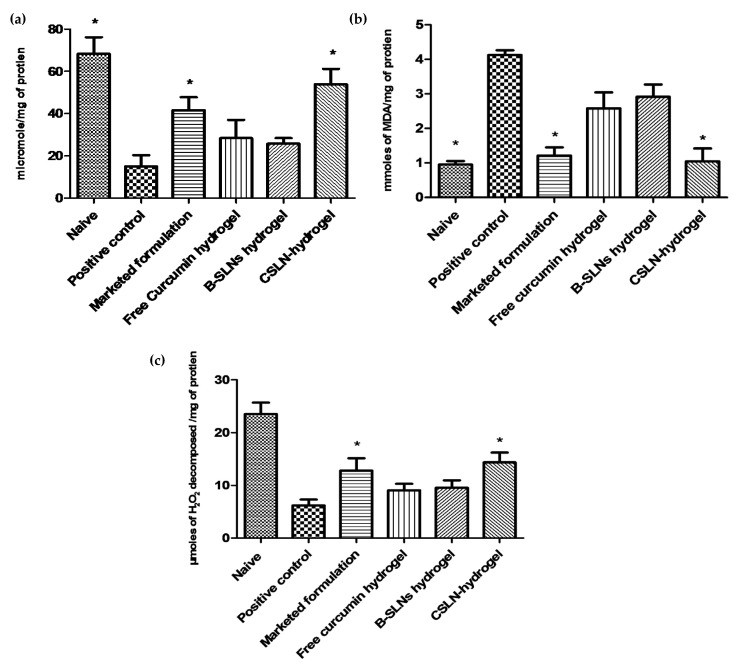
Effect of various treatments on (**a**) GSH levels, (**b**) LPO levels, and (**c**) catalase levels. Effect of various treatments such as blank (B) SLN hydrogel, free curcumin hydrogel, CSLN hydrogel, and the marketed formulation on antioxidant levels in skin homogenates. Data are represented as mean ± SD. Statistical significance was determined by ANOVA followed by Tukey HSD. CSLN hydrogel results in significantly better effect from all groups, except those marked with * (*p* < 0.001).

Presently, catalase and GSH levels increased, LPO decreased (Figure 8) and oxidative stress was attenuated significantly at the wound site in the CSLN hydrogel-treated group. A significant improvement in antioxidant levels, much better than the free curcumin hydrogel group, is attributed to the availability of curcumin at the wound bed in a more soluble, stable, permeable, and bioavailable form due to its encapsulation in the lipid core of SLNs, from which it is released slowly. It may also be noted that the CSLN hydrogel treatment completely attenuated the oxidative stress induced upon wounding and the values matched those for the naïve control.

A cascade of events including inflammation, proliferation, and migration of different types of cells are responsible for the healing of injured tissues [73]. Inflammation, which constitutes a part of the acute response, results in a coordinated influx of neutrophils at the wound site. These cells, through their characteristic “respiratory burst” activity, produce free radicals [74]. Wound-related non-phagocytic cells also generate free radicals by involving the non-phagocytic NAD(P)H oxidase mechanism [75]. Thus, the wound site is rich in reactive species resulting in oxidative stress leading to lipid peroxidation, and DNA breakage [76] contributing to impaired healing [7]. Further, injury also results in the loss of different enzymatic and non-enzymatic free radical scavengers which recover either partially or completely following healing.

#### 4.8.4. Effect of CSLNs Topical Application on TNF-α and VEGF

**Figure 9:**
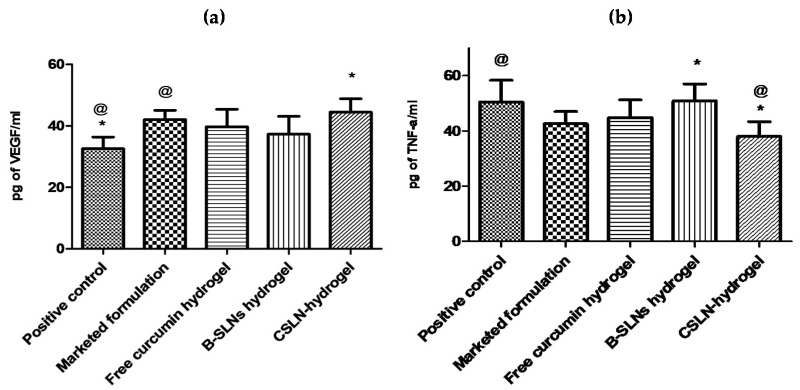
Effect of various treatments on (**a**) VEGF levels and (**b**) TNF-α levels. Effect of various treatments such as blank SLN hydrogel, free curcumin hydrogel, CSLN hydrogel, and the marketed formulation on TNF-α and VEGF levels in the skin homogenates. Data are represented as mean ± SD. Statistical significance was determined by ANOVA. The CSLN hydrogel group showed significant improvement (*, @) from the positive control (*p*< 0.05).

In the present study, the levels of VEGF increased in treatment groups in comparison to the positive control, with the CSLN hydrogel exhibiting VEGF levels higher than other groups (Figure 9a). One of the most important proangiogenic mediators is vascular endothelial growth factor (VEGF or VEGF-A), and sufficient VEGF levels are believed to be essential for proper wound healing. VEGF stimulates angiogenesis and influences wound closure and epidermal repair, granulation tissue formation, and the quality of repair—both in terms of the strength of the healed wound and the amount of scar tissue that is deposited [77]. Better and even neovascularization at the wound site in curcumin-treated rats in comparison to the positive control using CD31 staining has been reported [78]. Further, topical application of curcumin was shown to significantly upregulate the mRNA and protein expression of VEGF and TGF-β_1_ until day 14 [78]. Similarly, in a recently reported in vitro study [79], a significant increase in the number of formed tubules, the upregulation of VEGF expressions and the migration ability of high glucose-induced human umbilical vein endothelial cells was observed when the latter were co-incubated with culture medium from LPS-stimulated macrophages with curcumin-loaded chitosan nanoparticles and free curcumin. The results indicated enhanced angiogenesis via the inhibition of macrophage-induced inflammation by curcumin-loaded chitosan nanoparticles and free curcumin.

The levels of TNF-α were reduced significantly (*p* < 0.05) in the skin samples upon treatment with the CSLN hydrogel on day 11 (Figure 9b). The free curcumin hydrogel and the marketed formulation also showed an effect, but the levels were significantly better for the CSLN hydrogel. A significant reduction in the levels of TNF-α on day 7 and day 14 in curcumin-treated streptozotocin-induced diabetic rats in comparison to the control is also reported [17]. Furthermore, it has been [80] found that curcumin treatment increased the expression of TNF-α mRNA and protein levels compared to the control group in the early phase of wound healing, as shown by the PCR array and immunohistochemistry. Further, decreased TNF-α levels were reported at days 3 and 7 in the curcumin-treated group, which remained high in the control group until day 12.

### 4.9. Histology and Immunohistochemistry

**Figure 10:**
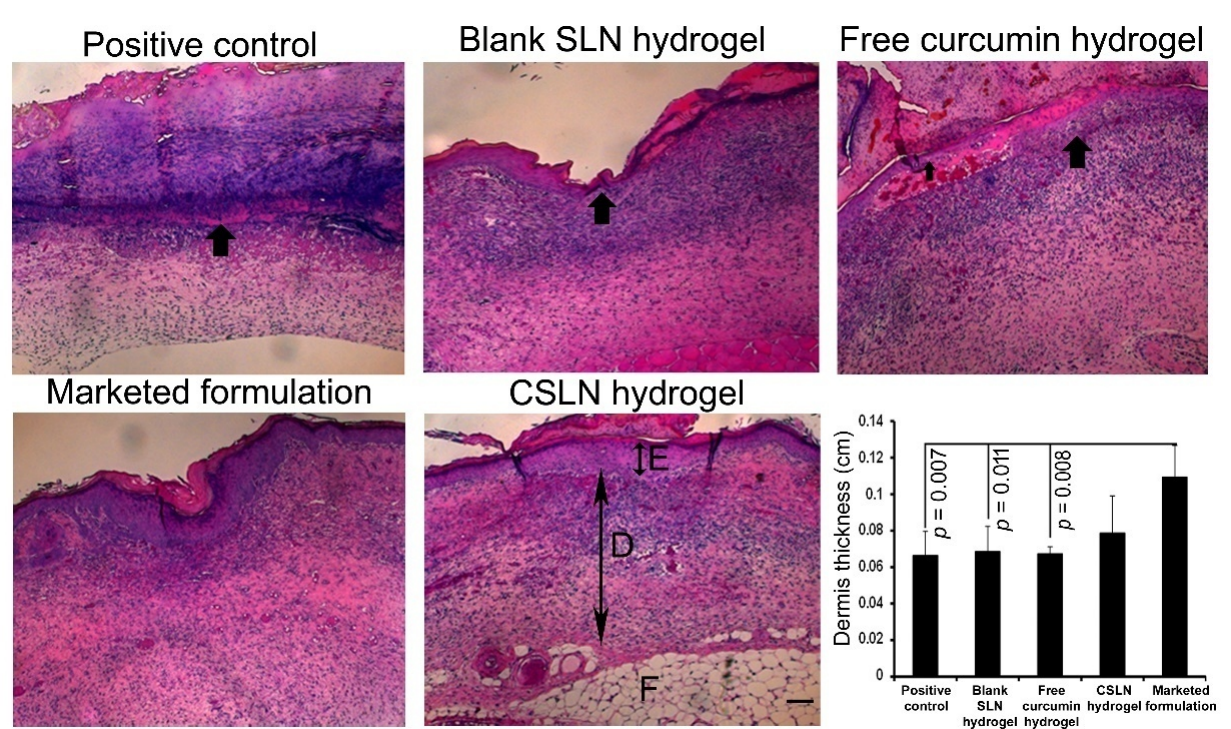
Impact of the CSLN hydrogel on full-thickness excisional wound healing. Representative images of histology of skin wound on day 11 in different treatment conditions (H&E stain) showing the epidermis (E, small double arrow) and dermis (D, large double arrow). Scale bar = 100 cm. Thick black arrows indicate the absence of epidermis and thinner dermal thickness in the positive control, blank SLN hydrogel, and free curcumin hydrogel group of mice. Data are represented as mean ± SEM. Statistical significance determined by ANOVA followed by post-hoc Fisher’s LSD test.

On day 11, the wound histology suggested no epidermis appearance in the positive control, blank SLN hydrogel, and free curcumin hydrogel group of mice as compared to the visible epidermis in the CSLN hydrogel and the marketed formulation-treated group of mice (Figure 10).

**Figure 11:**
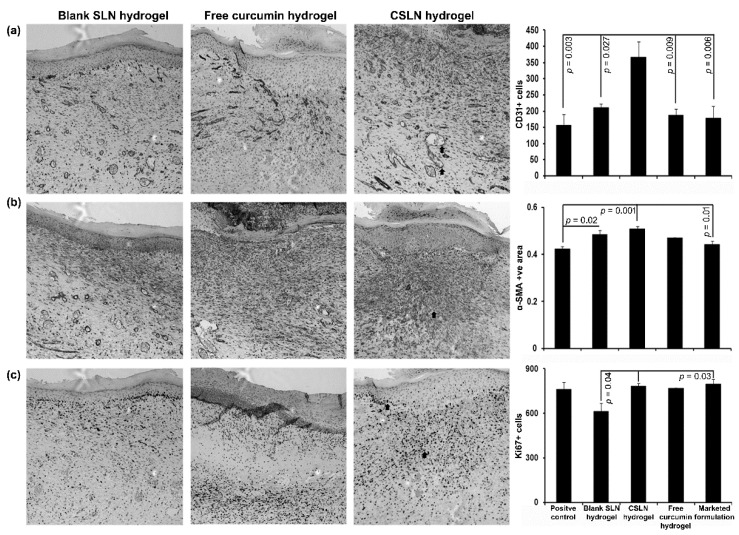
Effect of CSLNs on the angiogenesis (CD31), remodeling (α-SMA), and proliferation (Ki67) in skin wound after healing at day 11. Representative images of blank SLN hydrogel, free curcumin hydrogel, and CSLN hydrogel are shown for different staining’s (top to bottom and left to right, respectively). Serial sections were stained separately with anti-CD31 (**a**), anti-α-SMA (**b**), and anti-Ki67 (**c**), and visualized with a 10X objective. The number of +Ve cells/area obtained by ImageJ analysis were compared between all the groups (right panel, top to bottom). Data are represented as mean ± SEM. Statistical significance was determined by ANOVA followed by Tukey HSD. Arrows indicate +Ve cells for respective staining.

The wound cross-sections also revealed a thinner dermis (*p* < 0.01) in the positive control, blank SLN hydrogel, and free curcumin hydrogel groups and comparable to the CSLN hydrogel (*p* > 0.05) in comparison to the marketed group. Furthermore, a significant increase was observed in the number of CD31+ cells (ANOVA, F = 6.886; *p* = 0.002) in the CSLN hydrogel-treated mice (Figure 11a). CD31+ cells are an indicator of angiogenesis [81]. The alpha-SMA+ area (ANOVA, F = 7988; *p* = 0.001, Figure 11b), and Ki67+ cell (ANOVA, F = 3.811; *p* = 0.025) number was also more in the CSLN hydrogel group as compared to other groups (Figure 11c). Thus, the CSLN hydrogel induces cellular changes thereby helping in skin wound healing significantly.

### 4.10. Antimicrobial Activity of CSLNs

The CSLN hydrogel (0.52% *w*/*w* curcumin) showed effects (wound contraction) similar or better than the marketed formulation (Soframycin^®^), which contained the antibiotic framycetin, at almost double the concentration (1%, *w*/*w*) with respect to curcumin; hence, it was considered suitable to evaluate the antimicrobial activity of CSLNs. The effects were assessed against planktonic and biofilms of *Staphylococcus aureus* 9144, which is a common wound pathogen [82].

#### 4.10.1. Effect against Planktonic Cells

The MIC value obtained for CSLN was 64 µg/mL, while the free curcumin (aqueous suspension) did not show any inhibition even at a concentration as high as 2 mg/mL. However, when curcumin was dissolved in DMSO, it showed an MIC of 32 µg/mL (Table 10). The use of DMSO addresses the inherent problem of the solubility as well as the permeability (DMSO is a permeation enhancer) of curcumin.

However, it may be noted that DMSO cannot be used in clinical settings as this solvent is not approved by the FDA (only approved for intravesical administration). Presently, CSLNs also address solubility and permeability issues of curcumin; however, they exhibit a controlled (zero-order) release of curcumin, when about 25% of the loaded curcumin is released after 24 h (Figure 5) which reaches to about 50% after 48 h. So, at times when the antimicrobial effect was studied, i.e., at 24 h only 25% (1/4th) of the curcumin was released from the SLN dispersion, while the MIC is only double (64 versus 32 µg/mL). This better-than-expected effect of SLNs of curcumin could be due to their easier diffusion into the cellular membrane of *Staphylococcus aureus* 9144 due to their small particle size, resulting in a larger surface area-to-mass ratio thereby a greater interaction with cells [83]. Several workers [84,85,86] have demonstrated a relationship between the in vitro release of the drug and its antibacterial efficacy where at early time points SLNs were less effective, but at the later times SLNs were much more effective than their respective solutions. No inhibition was observed in the case of blank SLNs.

The antimicrobial property of curcumin involves its interaction with the essential cell division-initiating protein filamenting temperature-sensitive mutant Z(FtsZ). The latter is a protein involved in bacterial cell division and is the first protein to appear at the impending site of division [87]. Molecular docking [88] demonstrated that the oxygen molecules of phenol and methoxy functional groups (linked to phenolic rings) and two carbonyl groups of curcumin interact with the catalytic site of FtsZ by forming hydrogen bonds, and potentially non-specific hydrophobic interactions.

#### 4.10.2. Effect against Biofilm Formation and Mature Biofilms

The antimicrobial effect of the prepared CSLNs against *Staphylococcus aureus* ATCC 9144 biofilms is given in Table 11. The CSLNs inhibit biofilm formation at 512 µg/mL and the MIC against mature biofilm was found to be 2048 µg/mL.

Although curcumin solution in DMSO inhibited biofilm formation at 64 µg/mL, it did not show any effect against mature biofilms as curcumin was precipitated from its solution in DMSO at concentration above 256 µg/mL. Hence, the poor solubility of curcumin compromised its efficacy in the case of both curcumin suspension as well as curcumin solution in DMSO. However, as discussed earlier, the high solubility and nano size of CSLN allowed for its better interaction at the cell surface, resulting in its internalization into the microbial cytoplasm due to less spatial hindrance. Additionally, the nano size allows for a greater surface area to interact with the microorganisms or other biological components, resulting in better penetration and interaction with the biofilm matrix and higher uptake by the cells.

## 5. Conclusions

In the present study, solid lipid nanoparticles with/containing curcumin with a high payload (15%), without using any organic solvent, were prepared, and optimized to overcome issues such as low solubility and stability associated with curcumin, hampering its clinical translation. The CSLNs were found to be safe, stable, including photostable, and autoclavable (sterility is an important consideration for wound dressings), and resulted in the extended and controlled release of curcumin with significantly improved antibacterial activity. This is the first report establishing the effect of curcumin (CSLNs) on the disruption of mature biofilms of *S. aureus* an established skin pathogen. We demonstrated that CSLNs significantly accelerated wound closure by comprehensive healing which included downregulation of the inflammatory response and oxidative stress, expedited re-epithelialization, angiogenesis, and improved granulation tissue formation. This led to accelerated wound closure within a period of 11 days, while the other groups showed delayed healing. These results suggest that CSLNs are promising, particularly for wound healing activity because of their enhanced solubility, stability, and sustained release of curcumin.

## Figures and Tables

**Table 1:** Designed formulations for the evaluation of CSLNs using the Taguchi design.

No.	Stirring Speed (rpm)	Stirring Time (min)	No. of Cycles	Tween 80 (% *w*/*w*)	PEG600 (% *w*/*w*)	Lipid (% *w*/*w*)	Phospholipon 90G (% *w*/*w*)	Particle Size (nm)	Entrapment Efficiency (%)	PDI
1	6000	5	3	8	5	3	0.4	694.1	45.6	0.256
2	6000	5	3	12	8	5	1	349.9	76.8	0.132
3	6000	10	6	8	5	5	1	501.9	50.6	0.275
4	6000	10	6	12	8	3	0.4	138.4	74.1	0.109
5	10,000	5	6	8	8	3	1	378.3	60.2	0.186
6	10,000	5	6	12	5	5	0.4	214.5	63.7	0.140
7	10,000	10	3	8	8	5	0.4	416.0	64.8	0.218
8	10,000	10	3	12	5	3	1	233.8	61.5	0.174

**Table 2:** Designed CSLN formulations and their response parameters using CCD.

No.	Tween 80% *w*/*w* (X_1_)	PEG 600% *w*/*w* (X_2_)	Particle Size (nm) (Y_1_)	Entrapment Efficiency (%) (Y_2_)
1	8	8	351.3	60.3
2	10	4.37868	400.3	47.6
3	12	8	150.3	76.8
4	7.17157	6.5	559.3	49.6
5	10	6.5	260.3	67.5
6	10	8.62132	200.3	71.9
7	10	6.5	255.6	67.5
8	10	6.5	262.3	63.8
9	8	5	598.6	45.9
10	12	5	220.3	69.5
11	10	6.5	270.3	65.1
12	10	6.5	250.6	64.9
13	12.8284	6.5	190.3	73.9

**Table 3:** Various formulations prepared for identifying and screening the highest and lowest concentrations of independent variables.

No.	Surfactant/Co-Solvent Type				Drug (%)	Manual Observation
	A	B	C	D		
F1	8.9	-	-	-	0.6	Settling of curcumin on keeping overnight. Crystals of curcumin seen in microscope.
F2	8.9	5	-	-	0.6	Settling of curcumin on keeping overnight. Crystals of curcumin seen in microscope.
F3	8.9	8	-	-	0.6	No settling of curcumin but crystals of curcumin were seen under microscope.
F4	8.9	-	5	-	0.6	Settling of curcumin observed.
F5	8.9	-	8	-	0.6	No settling of curcumin but crystals of curcumin were seen under the microscope. Gelucire was expelled out.
F6	8.9	5	5	-	0.6	No settling of curcumin but crystals of curcumin were seen under the microscope. Gelucire was expelled out.
F7	8.9	5	8	-	0.6	No settling of curcumin but crystals of curcumin were seen under microscope. Gelucire was expelled out.
F8	8.9	-	-	5	0.6	Settling of curcumin observed.
F9	8.9	-	-	8	0.6	Settling of curcumin observed.
F10	8.9	5		8	0.6	Crystals of curcumin observed under the microscope.
F11	8.9	5		10	0.6	Crystals of curcumin observed under the microscope.
F12	8.9	8		10	0.6	Crystals of curcumin were not observed under the microscope.
F13	12	5	-	-	0.6	Crystals of curcumin observed under the microscope.
F14	12	8	-	-	0.6	Crystals of curcumin were observed under the microscope.
F15	12	-	-	5	0.6	Crystals of curcumin observed under the microscope.
F16	12	-	-	8	0.6	Crystals were not observed under microscope until 4 weeks.

A = Tween 80, B = PEG 400, C = Gelucire14/44, D = PEG 600.

**Table 4:** Model summary statistics of the response surface model.

Response	Model							Lack of Fit	
	F-Value	Prob > F	Adeq. Precision	CV%	R^2^			F-Value	Prob > F
					R^2^	AdjsR^2^	PredR^2^		
Y_1_	357.71	<0.0001	55.512	3.69	0.9961	0.9933	0.9775	4.09	0.1037
Y_2_	34.54	<0.0001	18.634	4.07	0.9610	0.9332	0.7744	4.27	0.0974

**Table 5:** Validation of prepared formulation.

Checkpoint Conditions X1/X2	Y_1_ (nm)			Y_2_ (%)		
	Observed	Predicted	Error (%)	Observed	Predicted	Error (%)
12/8	156.6	165.5	5.38	74.6	76.5	2.46
10/6.5	232.6	259.8	4.05	63.5	65.8	3.44
12/5	212.9	226.8	6.17	69.4	66.0	−5.13
8/8	365.5	352.1	−3.79	65.3	61.4	−6.33
8/5	614.6	590.8	−4.02	41.6	43.8	5.12

**Table 6:** Stability studies on optimized CSLN formulation (*n* =3).

Temperature	Months	% Decrease		% Increase in Particle Size	PDI
		TDC	% Entrapment		
5 ± 3 °C	0	-	-	-	0.206 ± 0.016
	1	0.78 ± 0.25	0.260.06	0.03 ± 0.00	0.213 ± 0.041
	3	1.80 ± 0.10	0.48 ± 0.06	0.03 ± 0.00	0.242 ± 0.026
	6	3.75 ± 1.61	4.75 ± 0.10	2.08 ± 2.23	0.242 ± 0.020
	12	5.56 ± 1.01	7.36 ± 0.89	9.24 ± 1.40	0.278 ± 0.015
30 ± 2 °C; 65% ± 5% RH	0	-	-	-	0.227 ± 0.003
	1	1.26 ± 0.40	0.54 ± 0.10	4.87 ± 0.36	0.307 ± 0.014
	3	2.15 ± 0.57	1.48 ± 0.10	19.93 ± 1.88	0.313 ± 0.031
	6	4.25 ± 0.98	6.26 ± 0.94	52.17 ± 4.19	0.294 ± 0.047
	12	9.80 ± 1.07	10.5 ± 1.48	72.21 ± 2.90	0.352 ± 0.000

**Table 7:** Photostability studies on optimized CSLN formulation (*n* = 3).

Glassware	Days	Assay/TDC%		% Entrapment	% Change in Particle Size
		Free Curcumin	CSLNs	CSLNs	
Amber glass	0 day	100.00 ± 0	100.00 ± 0	100.00 ± 0	-
	10 days	78.31 ± 7.1	99.64 ± 0.02	99.82 ± 0.6	7.90
Transparent	0 day	100.00 ± 0	100.00 ± 0	100.00 ± 0	-
	10 days	65.51 ± 7.5	98.93 ± 0.05	99.03 ± 0.06	8.98

**Table 8:** Parameters before and after autoclaving (*n* = 6).

Autoclaving	Assay/TDC * (mg/mL)	Entrapment Efficiency * (%)	Particle Size (nm)	PDI	Zeta Potential *
Before	5.8 ± 0.2	75.55 ± 2.31	170.1 ± 26.6	0.143 ± 0.026	−9.67±1.47
After	5.7±0.3	74.24± 3.6	253.7± 28.0	0.182 ± 0.032	−9.50±1.86

* No significant change.

**Table 9:** Drug release models.

Model	Formulations					
	CSLNs	CSLN Hydrogel	Free Curcumin Suspension	Free Curcumin Suspension Hydrogel	Free Curcumin Solution	CurcuWin^®^
Zero order (r^2^)	0.986	0.984	0.889	0.986	0.936	0.893
First order (r^2^)	0.935	0.979	0.928	0.978	0.990	0.982
Higuchi (r^2^)	0.957	0.960	0.974	0.913	0.878	0.990
KorsmeyerPeppas model (r^2^)	0.698	0.667	0.641	0.703	0.692	0.682

**Table 10:** Antimicrobial effect of CSLNs against *Staphylococcus aureus* ATCC 9144.

No.	Agents	MIC (µg/mL)
1	Curcumin in DMSO	32
2	Curcumin suspension in CMC	No inhibition
3	CSLNs	64
4	Blank SLNs	No inhibition

**Table 11:** Antimicrobial effect of CSLNs against *Staphylococcus aureus* ATCC 9144 biofilms.

No.	Agents	MIC (µg/mL)
Biofilm Formation		
1	Curcumin solution in DMSO	64
2	Curcumin suspension in CMC	No inhibition
3	CSLNs	512
Mature Biofilms		
1	Curcumin solution in DMSO	No inhibition
2	Curcumin suspension in CMC	No inhibition
3	CSLNs	2048

## Data Availability

All relevant data is included in Supplementary File.

## Supplementary Materials

The following are available online at https://www.mdpi.com/article/10.3390/antiox10050725/s1. The following are available as attached file as “Supplementary Data”.
