# Supplementary material for: Systematic Development and Characterization of Novel, High Drug-Loaded, Photostable, Curcumin Solid Lipid Nanoparticle Hydrogel for Wound Healing
Source: Antioxidants (Basel). 2021 May 5;10(5):725. doi: 10.3390/antiox10050725 (PMC8148018; doi:10.3390/antiox10050725)
Supplement: Supplementary file 1 [file antioxidants-10-00725-s001.zip › antioxidants-1187238-supplementary.pdf]

Methods

S 1.2.1 Characterization

S 1.2.1.1 Determination of Total Drug Content (TDC)/Assay

Depending upon the solubility of Compritol®888 ATO, methanol: chloroform (1:1) was chosen as a solvent for disrupting the SLNs. 1 mL of CSLN (n=6 batches) was suitably diluted to 1000 times with chloroform: methanol solvent system. The obtained solution was analysed spectrophotometrically at λ_{max} 425 nm using corresponding blank. TDC was determined using the following equation:

$$Total\ drug\ content(\%) = \frac{Observed\ drug\ content}{Theoretical\ drug\ content} \times 100$$

S 1.2.1.2 Determination of Entrapment Efficiency (EE)

The EE of CSLNs of 12 different batches was determined using dialysis membrane. The membrane was soaked in double distilled water overnight before use. 1 mL of CSLN dispersion was placed in the pre-soaked dialysis bag tied at both the ends and dialyzed against methanol (100mL) at room temperature for 45 minutes. The amount of drug remaining in the dialysis bag was analyzed spectrophotometrically following appropriate dilution (1000 times) with methanol: chloroform (1:1) to calculate the entrapment efficiency using the following equation:

$$\text{Entrapment Efficiency } (\%) = \frac{Entrapped\ drug}{Total\ drug\ content} \times 100$$

S 1.2.1.3 Particle Size Analysis and Polydispersity Index

The mean diameter and PDI of CSLNs (n=6) were determined after appropriate dilution (10 times) with double distilled water using Delsa™ Nano C Particle Analyser (Beckman Coulter, USA).

S 1.2.1.4 Zeta Potential

The zeta potential of CSLNs (n=6) was determined after appropriate dilution (10 times) with double distilled water using Delsa™ Nano C Particle Analyser (Beckman Coulter, USA).

S 1.2.2 Preparation of CSLNs Hydrogel

Briefly 1.5% w/v Carbopol 934P was soaked in water overnight. For neutralizing the basic nature of Carbopol 934P, it was treated with 1-2 drops of triethanolamine the next day to get a uniform gel. For every 9 g of CSLNs, 1g of Carbopol 934P (150 mg Carbopol 934P soaked in 850 mg of water) gel was added to get 10g of CSLN hydrogel after thorough mixing.

S 1.2.3 Assay/Total Drug Content of the CSLN Hydrogel

TDC of CSLN hydrogel was measured by mixing its known quantity with sufficient distilled water and vortexing for 5 min. The obtained dispersion was diluted with chloroform:methanol (1:1), filtered and analysed spectrophotometrically at λ_{max} 425nm using respective blank.

S 1.2.4 Texture Analysis of CSLN Hydrogel

The mechanical property of CSLN hydrogel was determined using a software-controlled penetrometer, texture analyser (Stable Micro systems, Surrey, UK). Bottle containing formulation was kept in an ultrasonic water bath (maintained at 37 °C) for 20 min in order to remove air bubbles. The probe was compressed into the CSLN hydrogel at a defined rate of 2mm s^{-1}. Various mechanical parameters such as hardness, compressibility, adhesiveness and cohesiveness of the gel formulation were estimated.

S 1.2.5 Safety Studies

S 1.2.5.1 In Vitro Cytotoxicity Studies

In vitro cytotoxicity studies were performed using human epidermis model episkin™ irritation test 42 h study. Briefly, plates containing epidermis after treating suitably with CSLN, sodium lauryl sulphate (positive control) and vehicle (negative control) were shaken for 15 min at 300 rpm followed by measurement of IL-1 α in the incubation media using Quantikine®ELISA Human IL-1α/IL-1F1 Immunoassay (catalog Number DLA50 Catalog Number SLA50 Catalog Number PDLA50).

S 1.2.5.2 In Vivo Safety Studies

Safety assessment for topical application was approved by the Institutional Animals Ethics Committee, Panjab University, Chandigarh (PU/IAEC/S/16/98), India and performed as per the OECD guidelines (Acute dermal toxicity study: OECD guideline 404 and repeated dose 28- day dermal toxicity study: OECD guideline 410).

S 1.2.5.2.1 Acute Dermal Toxicity Study (OECD Guideline 404)

Adult albino rabbits (weight 1-2 kg) were used for the acute studies. Approximately 24 h before the test, fur was removed by closely clipping the dorsal area of the trunk of the animals after which hair were removed completely by applying a depilatory. Care was taken to avoid abrading the skin, and only animals with healthy, intact skin were used. A dose of 0.5g of CSLN hydrogel was applied to the test site. The testing was done initially using one animal. The test substance was applied to a small area (approximately 6 cm^2) of skin and covered with a gauze patch, which was held in place with a non-irritating tape. Access by the animal to the patch and ingestion or inhalation of the test substance was prevented by applying the patch near the neck area. The exposure period was 4 h, after which the patch was removed, and the animal was observed for the next 72 h for any signs of redness, erythema and oedema. Since no corrosive effect was observed with the first animal, the negative response was confirmed using two additional animals, each with one patch, for an exposure period of 4 h. After removal of the patch, the animals were similarly observed up to 72 h. After removal of the patches, all animals were examined for signs of erythema and oedema and scored (Table S1), and the response was scored at 60 minutes, 24, 48 and 72 h after removal of the patch. For the initial test in one animal, the test site was also examined immediately after the patch was removed. The animals were kept under observation for the next 14 days when they were observed every day regularly (OECD, 2015).

**Table S1.** Grading of skin reaction.	
**Erythema and Eschar Formation**	
No erythema	0
Very slight erythema	1
Well defined erythema	2
Moderate to severe erythema	3
Severe erythema (beef redness) to eschar formation preventing grading of erythema	4
**Oedema Formation**	
No oedema	0
Very slight oedema (barely perceptible)	1
Slight oedema (edges of area well defined by definite raising)	2
Moderate oedema (raised approximately 1 mm)	3
Severe oedema (raised more than 1 mm and extending beyond area of exposure)	4

S 1.2.5.2.2 Repeated Dose 28-Day Dermal Toxicity Study (OECD Guideline 410)

The individual weight of the animals (Wistar rats) was taken before the start of the study. Ten animals (5 male and 5 female) each with healthy skin were used for treatment while another set of 10 animals (5 male and 5 female) were used as control. The repeated dose 28-day dermal toxicity study was performed in accordance with the OECD guidelines (OECD TG 410). According to this, 1000mg/kg dose can be used if test substance is not expected to produce any toxic effects. Hence 1000 mg/kg was used presently, as curcumin is expected to be safe on topical application and SLNs are also reported as a safe topical system. CSLN hydrogel was applied uniformly over an area which was approximately 10 percent of the total body surface area (10.5 cm^2). The application was covered with a gauze patch, which was held in place with non-irritating tape. The application was repeated every day for 28 days. The animals were observed throughout the dosing period. General clinical observations were made each day before applying the dose. These included change in skin, fur, eyes, occurrence of secretion and excretion and autonomic activity (e.g. piloerection, unusual respiratory pattern) as well as change in gait, posture and response to handling as well as the presence of tonic or clonic movements, stereotypes (e.g. excessive grooming, repetitive circling) or bizarre behaviour (e.g. self-mutilation, walking backwards). Body weights were measured weekly (OECD, 1981).

Results and Discussion

S 2.1 Characterization

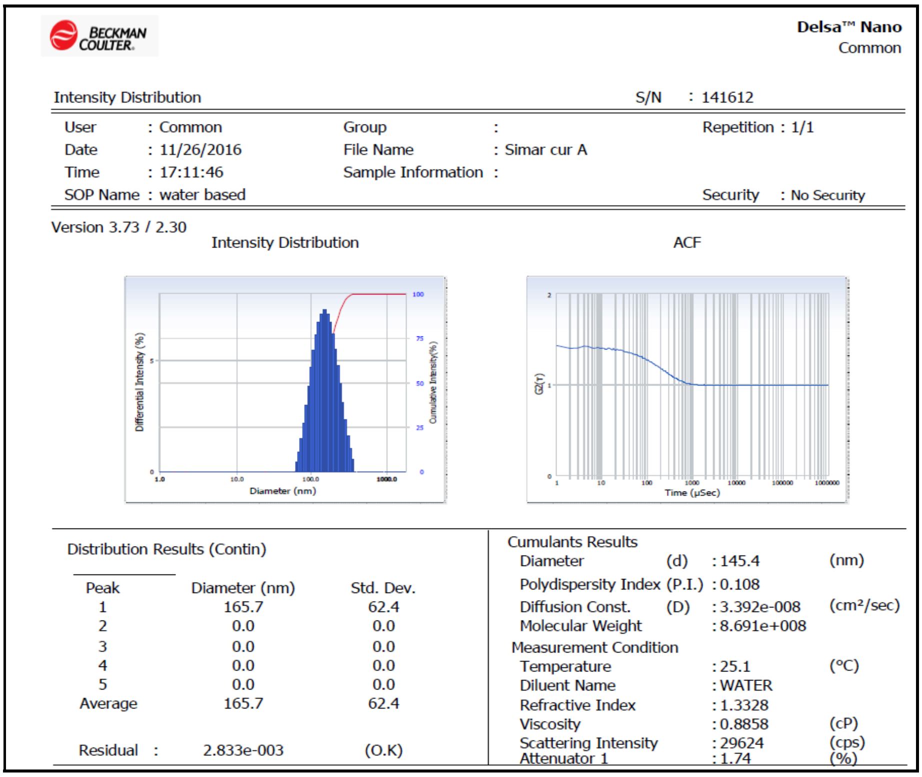

(a)

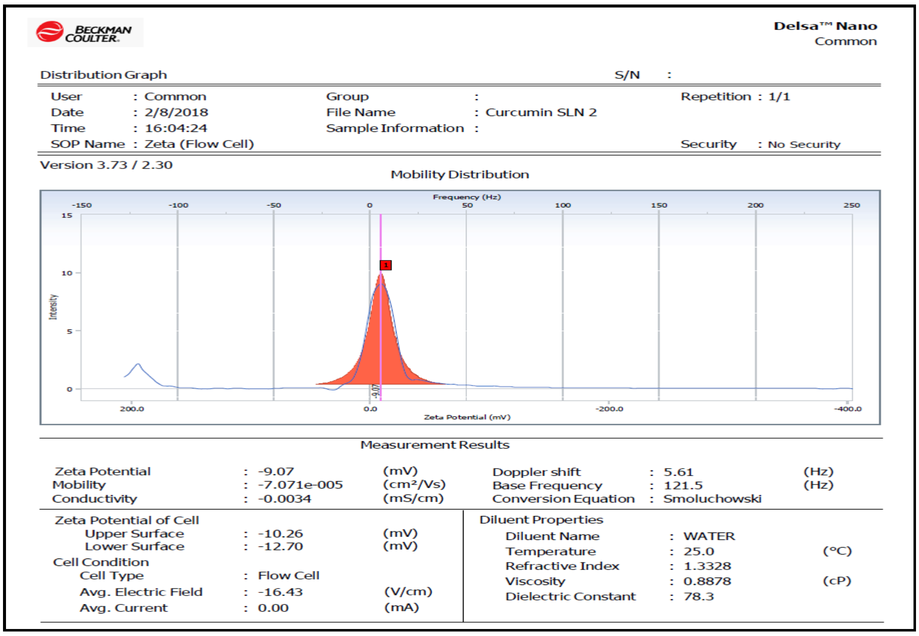

(b)

Figure S1. (**a**) Particle size of a representative optimized batch of CSLNs, (**b**) Zeta potential of the representative optimized batch of CSLNs.

S 2.1.1 Powder X-ray Diffraction (PXRD) Study

PXRD pattern of CSLNs (Figure S2a); blank SLNs (Figure S2b); free curcumin (Figure S2c); curcumin solution in PEG 600 (Figure S2d); curcumin, PEG 600 and Compritol® 888 ATO mixture (Figure S2e); and Compritol® 888 ATO (Figure S2f) are shown below. Curcumin showed sharp peaks at 2θ scattered angles of 8.78° and 17.16°, and some peaks of lower intensity at 10.64, 18.79. 23.43 and 29.51 indicating its crystalline nature (Figure S2c). In case of lyophilised CSLNs (Figure S2a), all these characteristic peaks are missing, indicating that curcumin is now present in an amorphous and thus soluble form within a lipid enclosure of SLNs. Further, XRD spectra of curcumin solution in PEG 600 (Figure S2d) and curcumin, PEG 600 and Compritol® 888 ATO physical mixture did not show any of the peaks depicted by free curcumin corroborating complete solubilization of curcumin in lipidic phase resulting in high entrapment efficiency. In addition, XRD spectra of Compritol® 888 ATO showed a reduced intensity in CSLNs (Figure S2a) and curcumin, PEG 600 and Compritol® 888 ATO mixture. This could be due to the intercalation of curcumin between parts of the lipid crystal lattice, leading to the lower crystallinity of the latter in CSLNs in comparison to bulk solid lipid. Similar observations are reported for curcumin incorporation within phopholipid-edge activator bilayer, resulting in the stabilization of the vesicular system (Zhang and Wang, 2016).

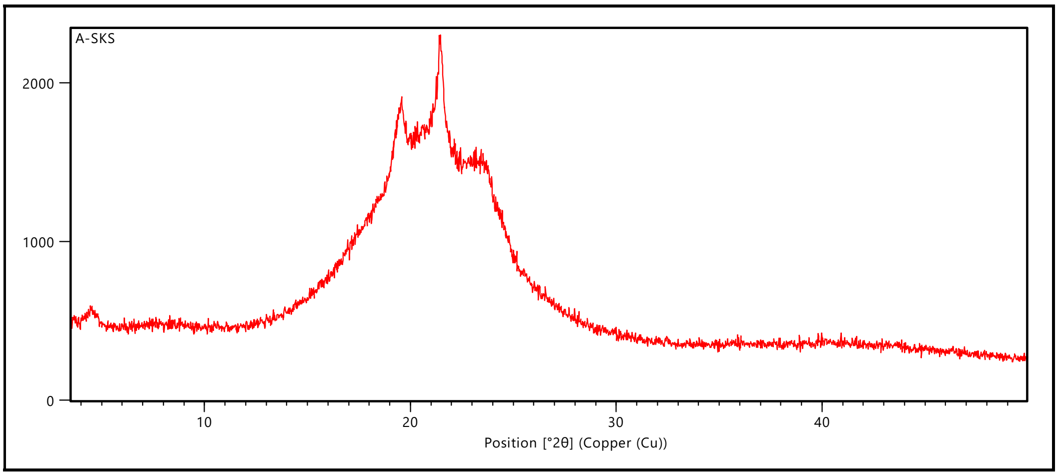

(a)

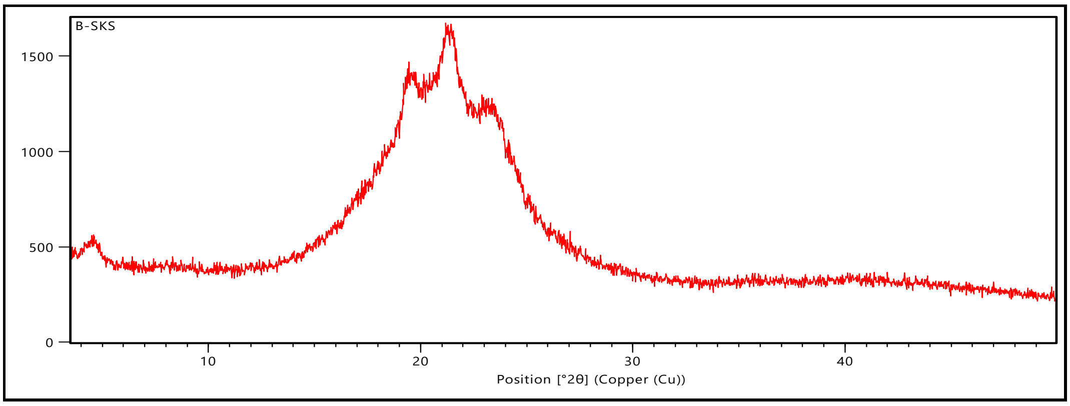

(b)

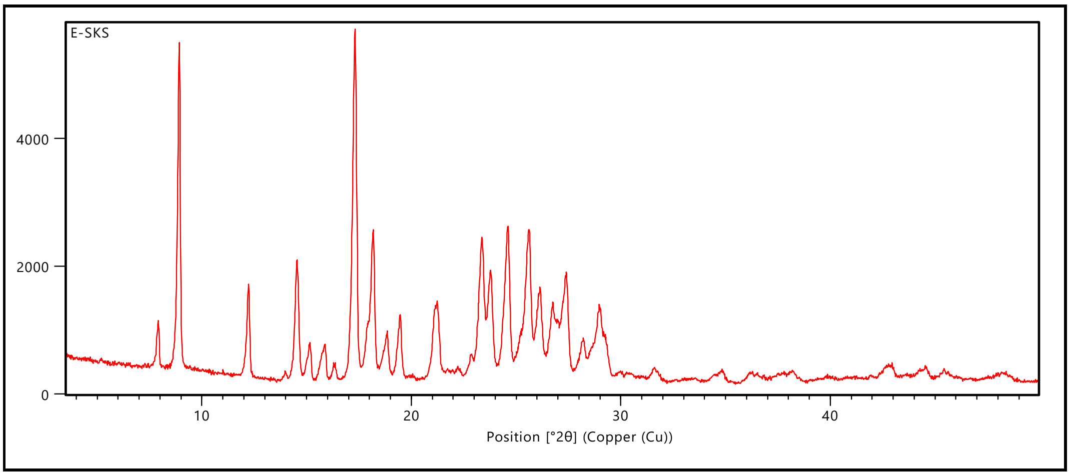

(c)

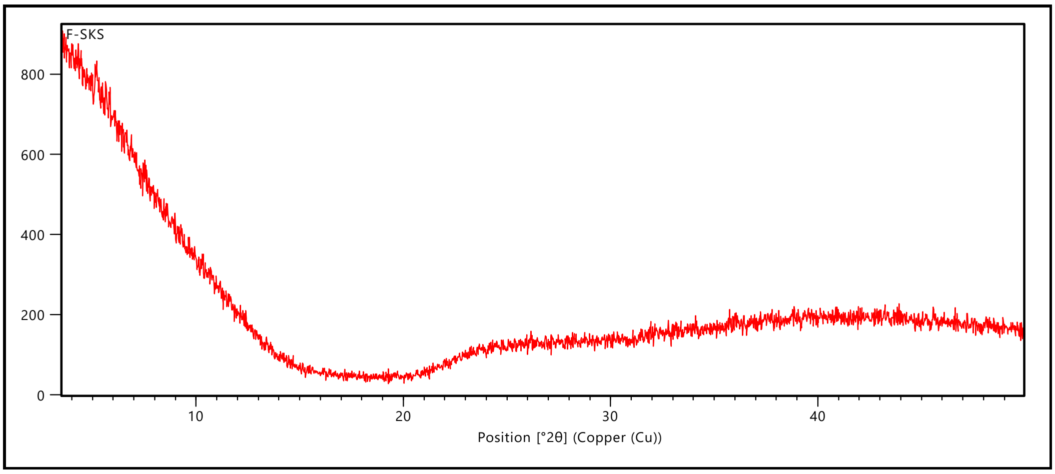

(d)

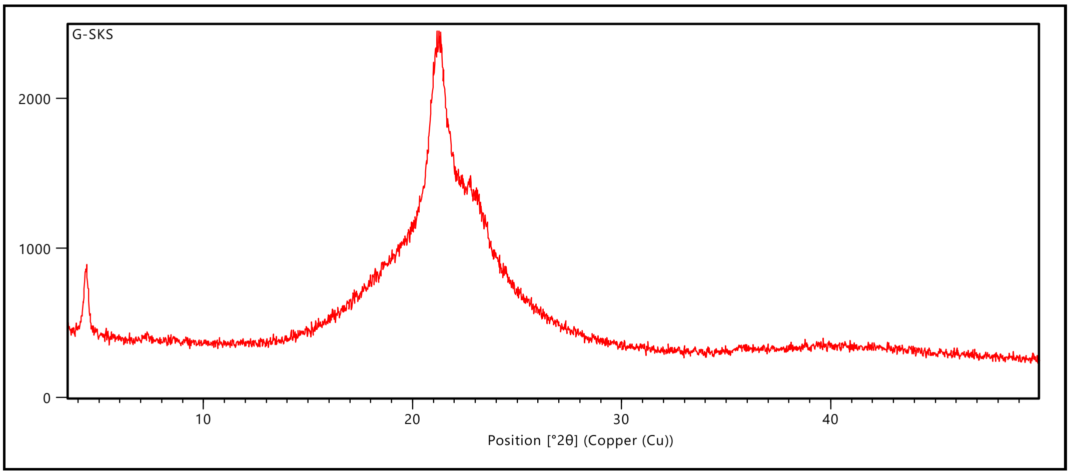

(e)

Figure S2. XRD spectra of (**a**) CSLNs, (**b**) blank SLNs, (**c**) free curcumin, (**d**) curcumin solution in PEG 600, (**e**) curcumin, PEG 600 and Compritol 888® ATO mixture.

S 2.1.2 Differential Scanning Calorimetery (DSC)

DSC is a thermoanalytical technique in which the differences in the amount of heat required to maintain the sample and reference at same temperature is measured as a function of temperature and time. In case of pure curcumin (Figure S3c), a melting endotherm appeared at 178.19°C (52.27 J/g) corresponding to its melting point at 180-183°C which disappeared in CSLNs thermogram. It could be attributed to change in the physical state of curcumin from crystalline to amorphous due to high entrapment within the lipid core of formed SLNs. This can be explained in that the lipid/ PEG 600 of the SLN formulation prevented crystallization of curcumin during the preparation process. The CSLNs show a sharp peak at 68.91°C and a broad endotherm starting from almost 78°C and continuing up to 116°C with a peak at 92.17°C (41.57J/g) (Figure S3a) is also observed. The Compritol 888® ATO thermogram (Figure S3d) had an endothermic peak at 73.11 °C, which is related to its melting point. This peak was down shifted to 68.91 °C in CSLNs. This phenomenon is described by the Gibbs–Thompson effect which states that the reduced particle size and increased surface area lead to a decrease in melting temperature compared to the bulk material (Perez, 2005). Furthermore, enthalpy of Compritol® 888 ATO decreased from 109.3 J/g to 19.32 J/g in CSLNs, which strongly confirms conversion of crystalline to amorphous form.

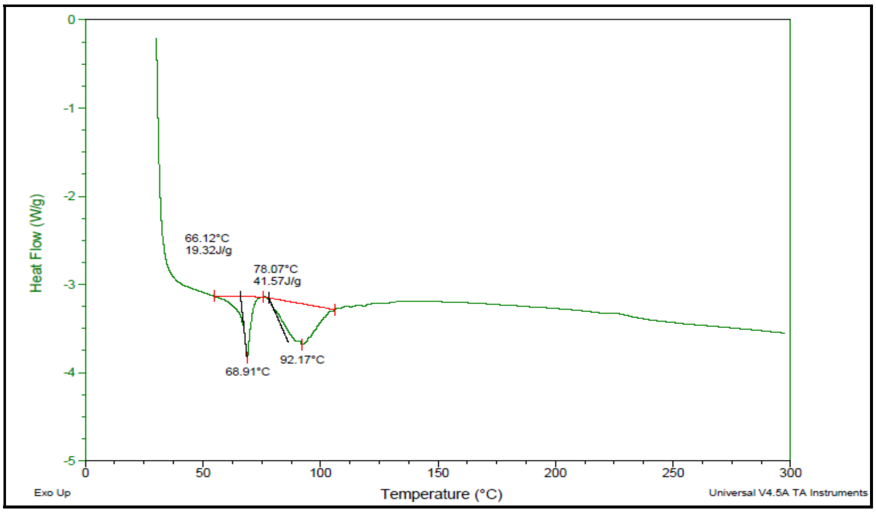

(a)

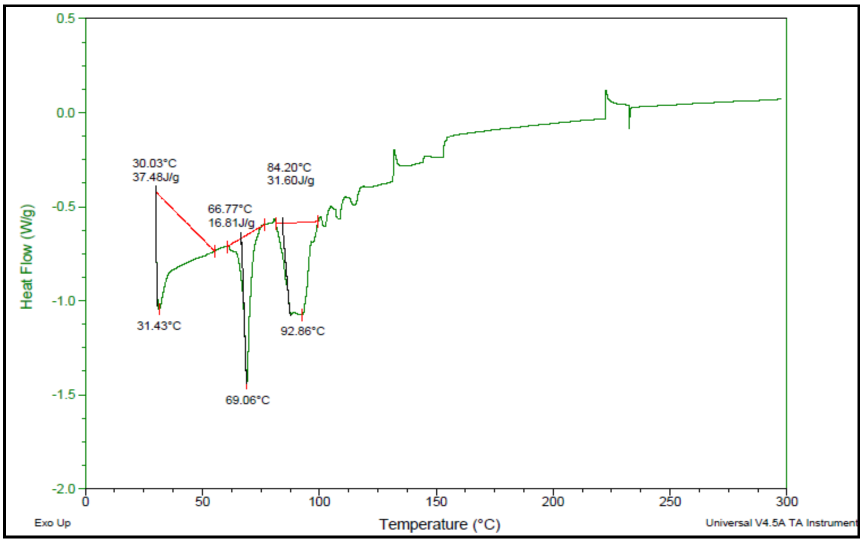

(b)

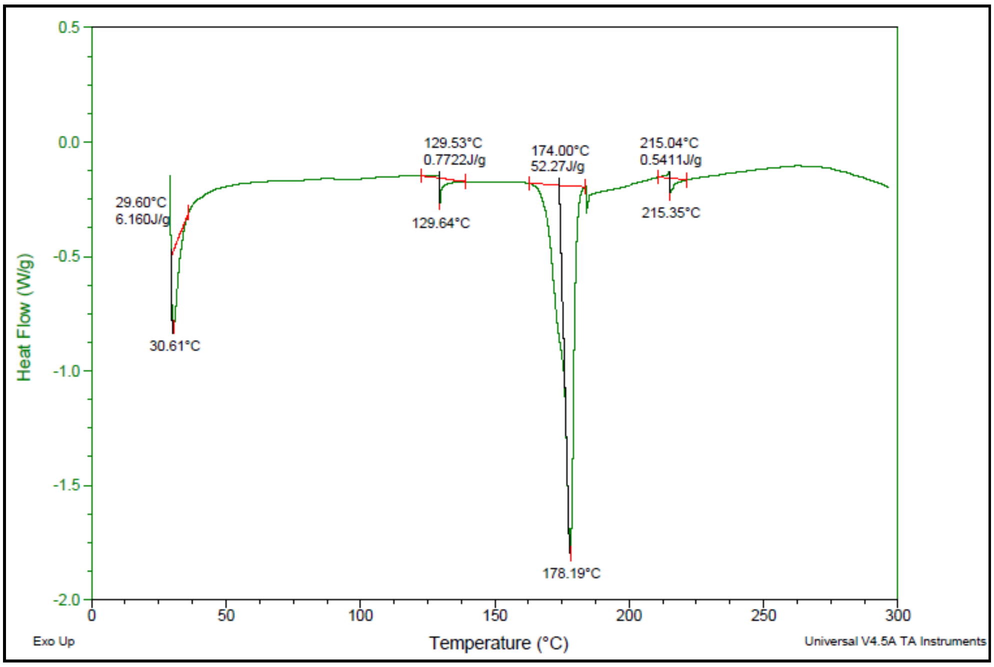

(c)

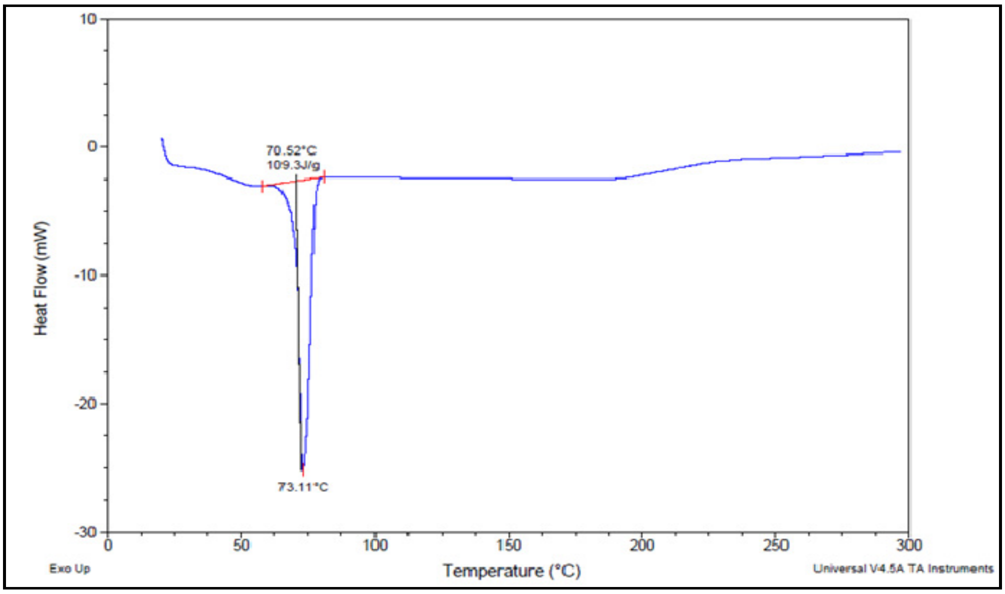

(d)

Figure S3. DSC of (**a**) CSLNs, (**b**) blank SLNs (**c**) free curcumin and (**d**) Compritol® ATO 888.

S 2.1.3 Fourier Transform Infra-Red (FTIR) Studies

For curcumin (Figure S4c), the broad absorption band at 3389.66 cm^{-1} and the sharp peak at 3507.78 cm^{-1} are attributed to the phenolic O-H stretching vibration. The bands at 1627.59 $cm^{-1}$and 1597.52 cm^{-1} are related to carbonyl group C = O and stretching vibrations of the benzene ring, respectively. The bands at 1427.63 cm^{-1} are related to olefinic bending vibration of C–H bound to the benzene ring of curcumin. The bands at 1277 cm^{-1} are due to aromatic C = O stretching. The 1511 cm^{-1} ring peak is assigned to the (C=O), while enol C-O peak was obtained at 1275.94 cm^{-1}, C-O-C stretching vibrations at 1027.25 cm^{-1} and benzoate trans-CH vibration at 959.43 cm^{-1} (Farnia *et al.*, 2016). The absence of characteristic curcumin peaks in solution of curcumin in PEG 600 (Figure

S4d) indicate solubilisation of curcumin. Further, absence of characteristic peaks of curcumin in the IR spectrum of the CSLNs (Figure S4a), indicate encapsulation of curcumin in the SLNs. Observation of broad absorption peak of OH at 3419 cm^{-1} in the FTIR spectrum of curcumin solution in PEG 600 (Figure S4d); in the mixture of curcumin, PEG 600 and Compritol® 888 ATO (Figure S4e); and in blank SLNs (Figure S4b) and CSLNs (Figure S4a) suggest that this peak is due to PEG 600 as also reported (Askari et al., 2019). This further complements the hypothesis that after addition of lipidic phase to aqueous phase, PEG 600 being highly hydrophilic leaves curcumin in the lipidic phase and moves to the aqueous phase.

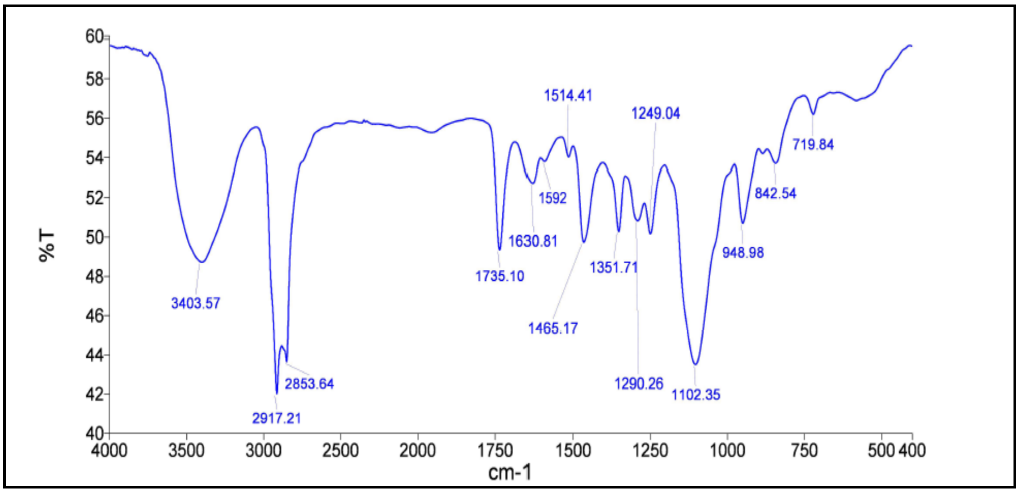

(a)

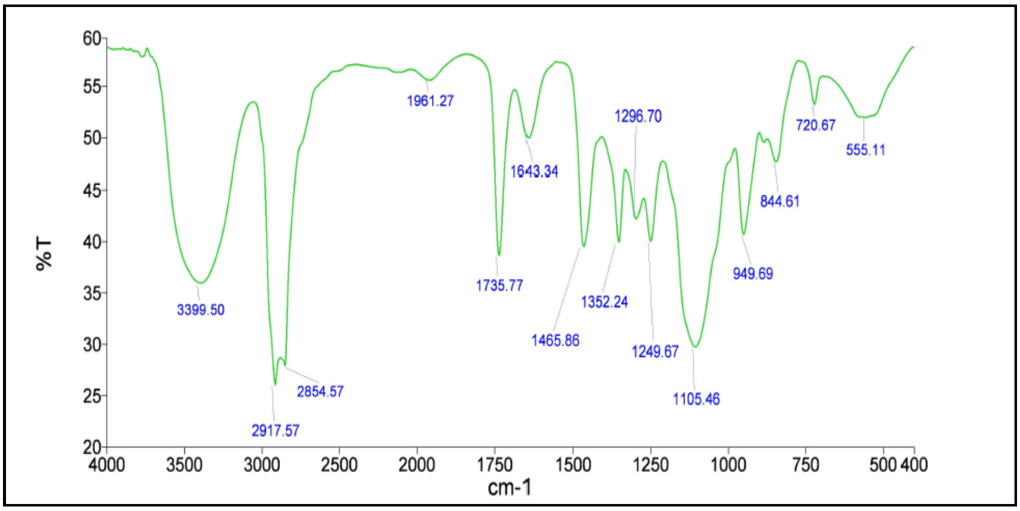

(b)

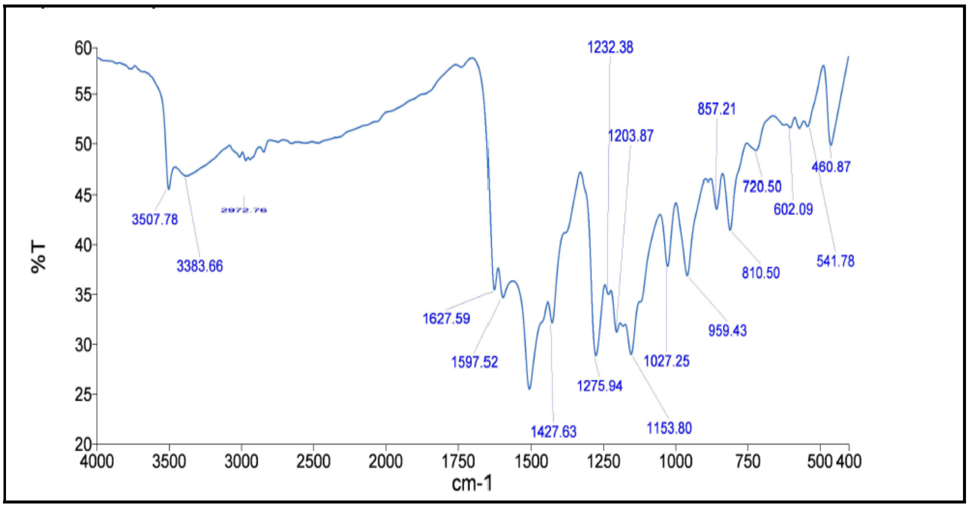
%T
cm-1
3507.78
3383.66
1627.59
1597.52
1427.63
1275.94
1232.38
1203.87
1153.80
1027.25
959.43
857.21
810.50
720.50
602.09
541.78
460.87

(c)

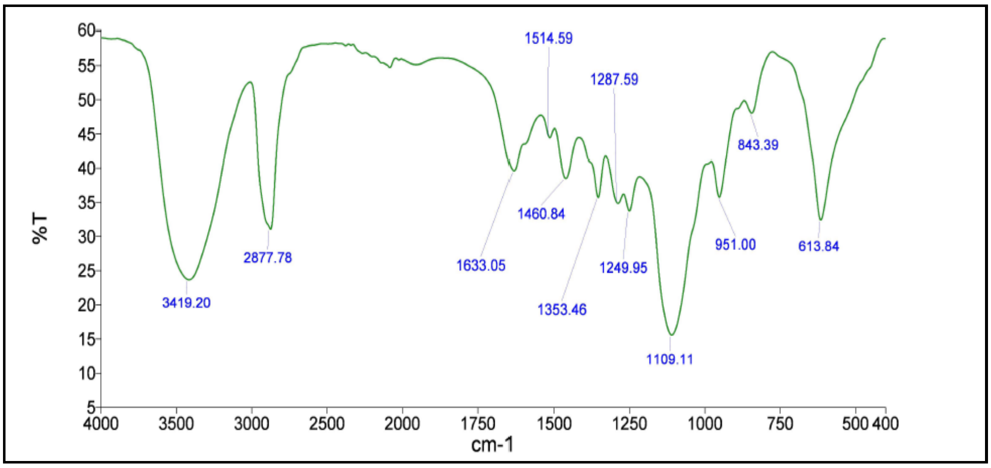
%T
cm-1
3419.20
2877.78
1633.05
1514.59
1460.84
1353.46
1287.59
1249.95
1109.11
951.00
843.39
613.84

(d)

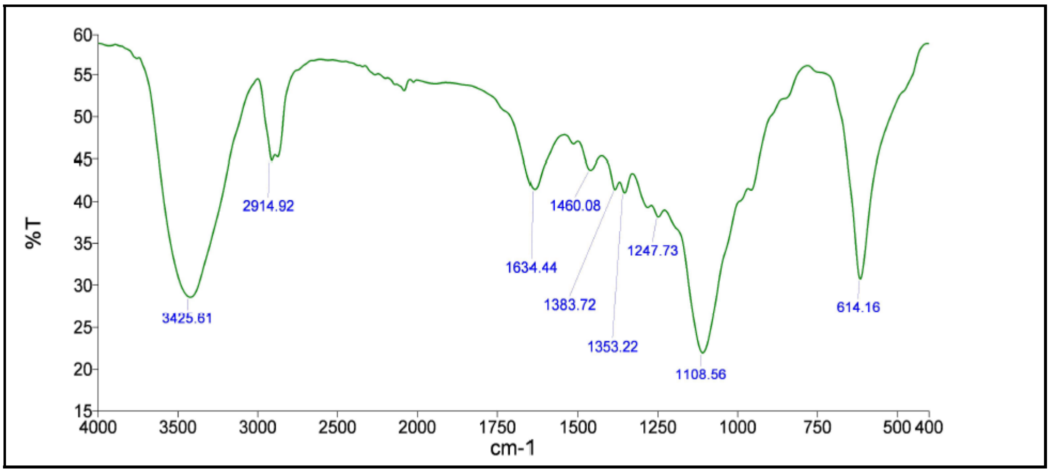
%T
cm-1
3425.61
2914.92
1634.44
1460.08
1383.72
1353.22
1247.73
1108.56
614.16

(e)

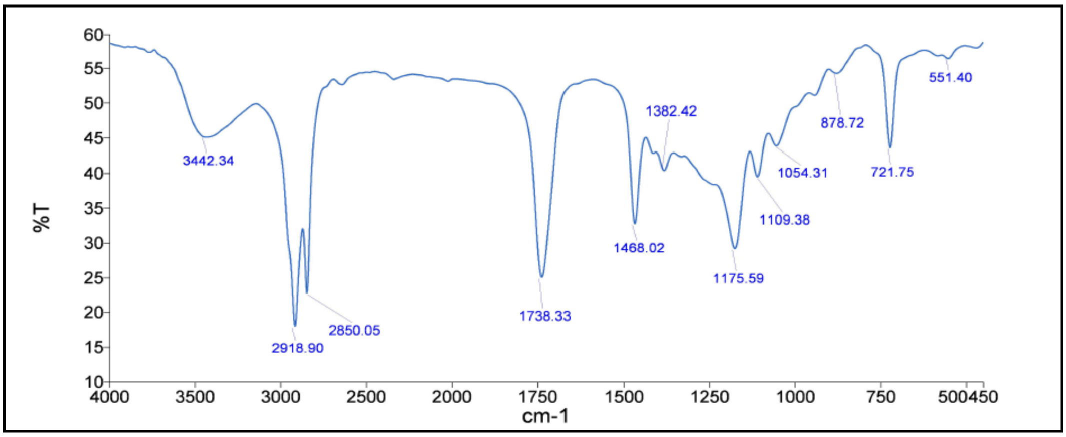

(f)

Figure S4. FTIR spectra of (**a**) CSLNs, (**b**) blank SLNs, (**c**) free curcumin, (**d**) solution of curcumin in PEG600, (**e**) curcumin, PEG 600 and Compritol® 888 ATO mixture and (**f**) Compritol® 888 ATO.

S 2.2 Assay/TDC of the CSLN Hydrogel

TDC of CSLN hydrogel was estimated to be 5.22 mg/g and matches to the actual content of 5.8 mg/mL of CSLNs.

S 2.3 Texture Analysis of CSLN Hydrogel

TPA (texture profile analysis) defines the mechanical parameters in terms of hardness, adhesiveness, cohesiveness, compressibility and consistency. The TPA graph and calculated mechanical properties of CSLN hydrogel are presented in Tables S2 and S3 and Figure S5a,b.

The hardness is defined as the maximum peak force during the first compression cycle. The hardness of CSLN hydrogel, which determines the ease of application on the skin, was 676.445, being acceptable for topical gel application. Adhesiveness is defined as the negative force area for the first compression cycle. It is the work required to overcome the attractive forces between the surface of the sample and the surface of the probe and it is related to bioadhesion (Jones et al., 1996). The adhesiveness value was calculated to be -0.229 g.sec. TPA also provides the information about the cohesiveness. Cohesiveness describes the ratio of the area under the force-time curve produced on the second compression cycle to that produced on the first compression cycle. The high value of cohesiveness provides full structural recovery following gel application. In present study, cohesiveness value was -3109.95g which is nominal for topical application (Karavana et al., 2009).

From the results of TPA experiments (Table S2) and spreadability test (Table S3), it can be concluded that CSLN hydrogel had suitable mechanical properties for topical application.

Table S2. Texture profile analysis.

Firmness (g)	Consistency (g.sec)	Cohesiveness (g)	Index of Viscosity (g.sec)
13789.47	235,960.97	−3109.95	−10,072.06

Table S3. Spreadability test.

Hardness (g)	Spreadability (g.sec)	Stickiness (g)	Adhesiveness (kg.sec)
676.445	899.543	−349.451	−0.229

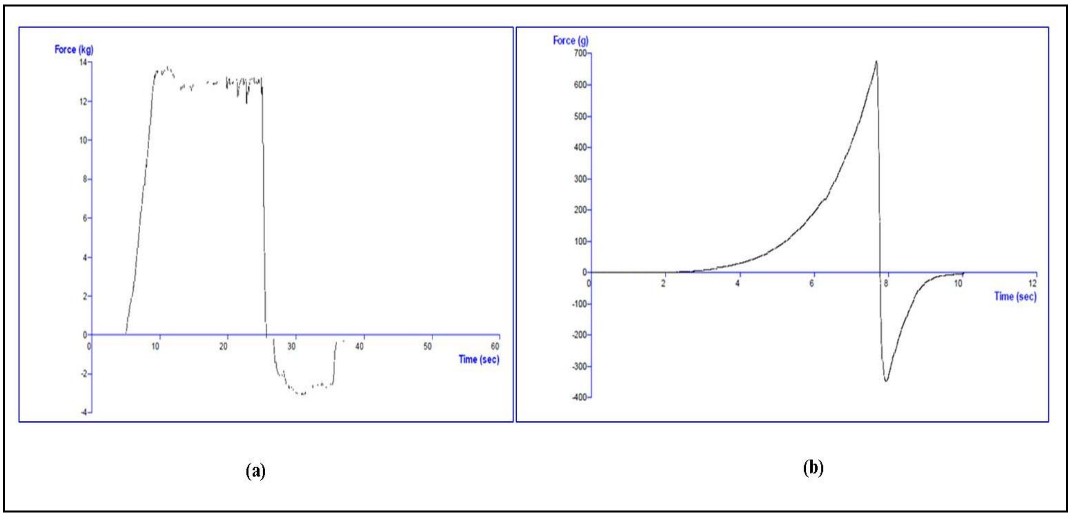

Figure S5. (**a**) Graph showed backward extrusion and (**b**) spreadability of CSLN hydrogel.

S 2.4 Safety Studies

Safety studies were done to study the potential irritation of surfactants or co-surfactants incorporated in CSLNs by determining their toxicity/ corrosion effects.

S 2.4.1 In Vitro Cytotoxicity Studies

The toxicity of nanomaterials is a common concern. While topical application of various nanoparticle formulations has not been associated with significant toxic effects or systemic absorption, compromise of the skin barrier (e.g., in the setting of acute or chronic wounds) may permit systemic absorption and internal organ accumulation of nanomaterials. Hence, in vitro cytotoxicity was done to asses the toxic nature of the prepared CSLNs. It was observed that there was no significant difference ($p<0.05$) between the % relative viability of negative control group and CSLN treated group confirming biocomaptible nature of CSLN and hence its suitability for topical application on wounds (Figure S6).

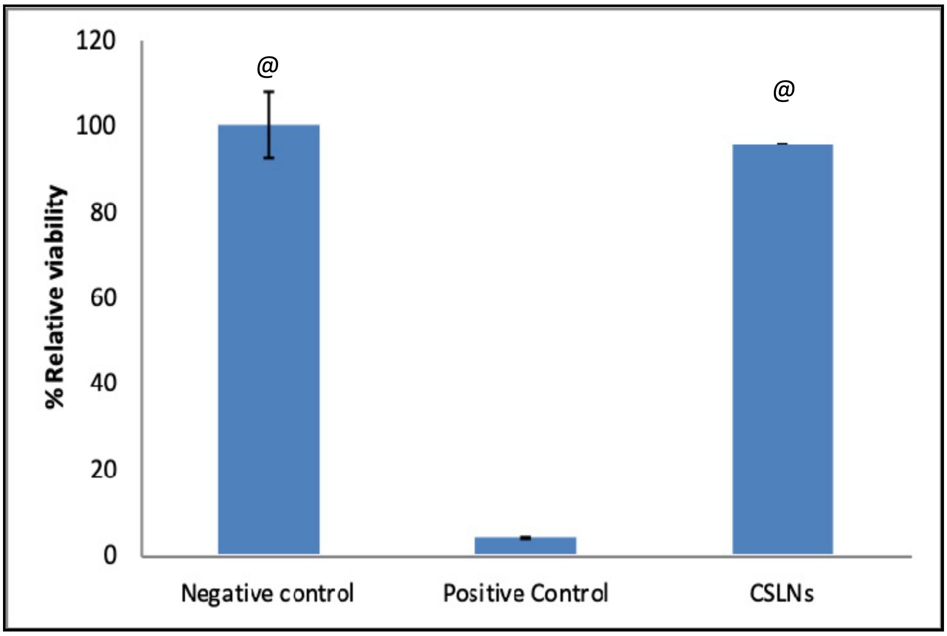

Figure S6. % Relative viability of CSLNs using human epidermis model Episkin™ irritation test. @ No significant difference was observed $p < 0.05$).

S 2.4.2 In Vivo Safety Studies

S 2.4.2.1 Acute Dermal Toxicity Studies (OECD TG 404)

All animals were examined for signs of erythema and oedema. The score for responses at 30 minutes, 60 minutes, and then at 24, 48 and 72 h after patch removal were zero, indicating that none of the treated animals showed any signs of adverse effects or toxicity, thus establishing safety of C-SLN hydrogel (Table S4). Figure S7 confirms absence of any observable adverse effects on the skin following topical application.

Table S4. Tabulation of scores for signs of irritation after various time interval.

Test substance: CSLN hydrogel									
**Test Animals: Albino rabbit**									
**Animal code**	**Signs**	**Dose mg/kg**	**0min**	**30min**	**60 min**	**1 hour**	**24hours**	**48hours**	**72hours**
S-1	Redness	500	0	0	0	0	0	0	0
	Erythema	500	0	0	0	0	0	0	0
	Oedema	500	0	0	0	0	0	0	0
S-2	Redness	500	0	0	0	0	0	0	0
	Erythema	500	0	0	0	0	0	0	0
	Oedema	500	0	0	0	0	0	0	0
S-3	Redness	500	0	0	0	0	0	0	0
	Erythema	500	0	0	0	0	0	0	0
	Oedema	500	0	0	0	0	0	0	0

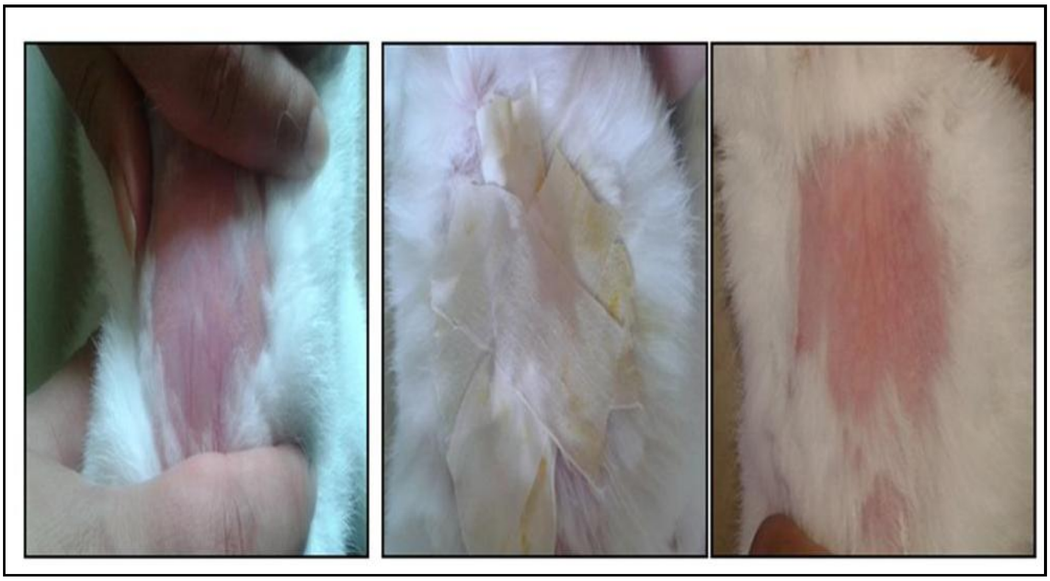

Figure S7: Skin of animals before (a), with (b) and after (c)the application of CSLN hydrogel – please label the pics as a, b and c

S 2.4.2.2 Repeated Dose Dermal Toxicity Studies (OECD TG 410)

Management of wounds requires repeated application of the product. Hence, repeated dose dermal toxicity studies were performed to assess the toxic nature of CSLN hydrogel on multiple dosing. No significant changes were observed in body weight of animals applied with 1000mg/kg of CSLN hydrogel as indicated in Table S5.

**Table S5.** Average body weight of animals in g (n = 10).					
**Groups**	**0 day**	**7th day**	**14th day**	**21st day**	**28th day**
CSLN hydrogel	165 ± 35	165 ± 35	165 ± 30	165 ± 35	165 ± 35
Naive	150 ± 45	150 ± 45	150 ± 45	150 ± 45	150 ± 45

Further, skin, fur, eyes, mucous membrane, behavioural pattern, salivation, sleep of the treated as well as control animals were found to be normal. No convulsion, lethargy, reduced activity and diarrhoea was observed with any of the treated animals. None of treated animal showed any adverse effects on the skin. From the histopathological studies Figure S8, it was observed that skin specimens after the application of CSLN hydrogel were normal.

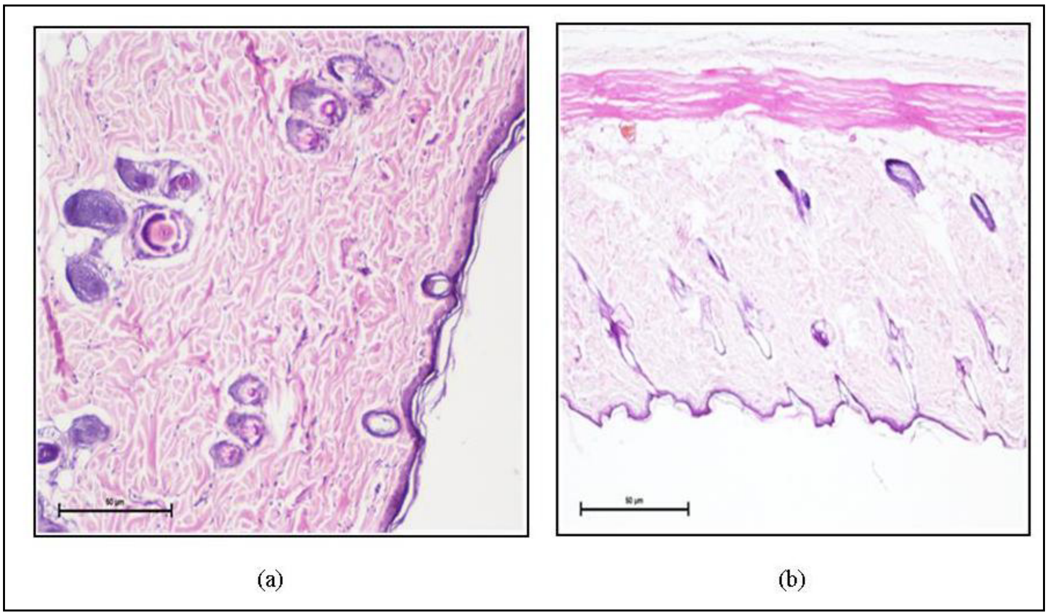

Figure S8. Histology of Skin Applied with CSLN hydrogel. (**a**) (40X) and (**b**) (100X).
